# Bidirectional communication between the gut microbiota and the central nervous system

**DOI:** 10.4103/NRR.NRR-D-25-00434

**Published:** 2025-09-03

**Authors:** Yingxian Liu, Tuoxian Tang, Hang Cai, Zhenjiang Liu

**Affiliations:** 1 National Engineering Laboratory for AIDS Vaccine, School of Life Sciences, Jilin University, Changchun, Jilin Province, China; 2 Department of Biology, University of Pennsylvania, Philadelphia, PA, USA; 3 Department of Pharmacy, The Second Hospital of Jilin University, Changchun, Jilin Province, China

**Keywords:** Alzheimer’s disease, amyotrophic lateral sclerosis, dysbiosis, gut microbiota, Huntington’s disease, inflammation, microbiota–gut–brain axis, neurodegenerative diseases, Parkinson’s disease, vagus nerve

## Abstract

In recent years, an increasing number of researchers have become interested in the bidirectional communication between the gut microbiota and the central nervous system. This communication occurs through the microbiota-gut-brain axis. As people age, the composition of the gut microbiota undergoes considerable changes, which are now known to play an important role in the development of many neurodegenerative diseases. This review aims to investigate the complex bidirectional signaling pathways between the gut and the brain. It summarizes the latest research findings on how the gut microbiota and its metabolites play critical roles in regulating inflammation, maintaining gut health, and influencing the development of neurodegenerative diseases such as Alzheimer’s disease, Parkinson’s disease, and amyotrophic lateral sclerosis. The review also analyzes the current clinical applications of gut microbiota-based treatments for neurological disorders, including fecal microbiota transplantation, probiotics, and prebiotics. Many studies show that the gut microbiota affects the brain in several ways. For example, it can produce substances such as short-chain fatty acids and activate inflammatory pathways. Studies involving animals and laboratory models have demonstrated that adjusting the gut microbiota can help improve behavior and reduce neurological problems. Recent metagenomic and metabolomics studies have shown that the microbiota plays a crucial role in maintaining the organism’s health. Microorganisms primarily colonize the gut and are involved in host nutrient metabolism, maintaining the structural integrity of the intestine, preserving the intestinal mucosal barrier, and modulating the immune system. The gut microbiota communicates with the brain through a bidirectional microbiota-gut-brain axis. The composition of the gut flora changes considerably with age, and ecological dysregulation has been recognized as one of the twelve most recent hallmarks of aging. Recent studies have linked these changes to a variety of age-related neurological disorders, including Alzheimer’s disease, amyotrophic lateral sclerosis, Parkinson’s disease, multiple sclerosis, and Huntington’s disease. Specifically, the gut microbiota influences the brain through the production of key metabolites such as short-chain fatty acids and the activation of inflammatory and other relevant signaling pathways. In preclinical studies, targeted modulation of the gut microbiota, through methods such as fecal microbiota transplantation, probiotics, and prebiotics, has demonstrated potential in improving host behavioral outcomes. Therefore, gut microbiota-based treatments offer new hope for the treatment of nervous system diseases. However, due to the complexity of the gut microbiota and the potential adverse reactions associated with these therapies, researchers need to carefully assess their safety and efficacy before widespread clinical application.

## Introduction

Trillions of microbes inhabit the human body, particularly in mucosal tissues such as the gut (You et al., 2025). The microbial population in the gut is considerably denser than in other bodily regions (Sender et al., 2016; Thursby and Juge, 2017), comprising approximately 100 distinct species. The gastrointestinal microbiota, which includes bacteria, archaea, and eukaryotes, has co-evolved with its host over millennia to establish a mutually beneficial relationship (Bäckhed et al., 2005). Remarkably, this microscopic ecosystem is estimated to contain **~**10^14^ individual microbes. Recent evidence suggests that microbial numbers are roughly equivalent to human cell counts (Sender et al., 2016), although earlier estimates proposed a tenfold greater ratio (Gill et al., 2006). The predominant phyla in the gut include *Bacteroidetes*, *Firmicutes*, *Actinobacteria*, and *Proteobacteria* (Korf et al., 2022). Colonization of the gastrointestinal tract begins at birth, with microorganisms acquired from the mother and the surrounding environment (Thursby and Juge, 2017).

The gut microbiota protects against pathogens by promoting intestinal epithelium regeneration (Li et al., 2025; Zhang et al., 2025a), strengthening barrier functions, competing for resources, producing antimicrobial substances such as hydrogen peroxide, and creating an acidic environment that inhibits pathogenic bacteria (Thursby and Juge, 2017). It also plays a vital nutritional role by fermenting complex carbohydrates (e.g., cellulose, pectin, and starch) and proteins to produce short- and branched-chain fatty acids, which serve as essential energy sources for colon cells (den Besten et al., 2013). Lactic acid bacteria synthesize vitamin B12, a nutrient that neither plants nor animals can produce (LeBlanc et al., 2013). In parallel, *Bifidobacteria* serve as primary producers of folate involved in DNA metabolism and generate essential nutrients such as vitamin K and the B-complex vitamins (Pompei et al., 2007). Clinical findings indicate that patients with inflammatory depression exhibit gut microbiota imbalances, including increased *Bacteroides*, reduced *Clostridium*, and dysregulated short-chain fatty acid-producing species characterized by abnormal butyrate metabolism (Liu et al., 2024a). Animal studies further demonstrate that transplanting fecal microbiota from these patients induces depressive- and anxiety-like behaviors in recipient mice, along with elevated peripheral and central inflammatory markers. Notably, supplementation with *Clostridium butyricum* restores microbial composition, reduces inflammatory parameters, and exerts antidepressant effects (Liu et al., 2024a). A recent study has confirmed that gut microbiota and its metabolites influence central nervous system (CNS) function by modulating the brain’s biochemical microenvironment and blood–brain barrier (BBB) permeability (Mayerhofer et al., 2017).

**Figure 1:**
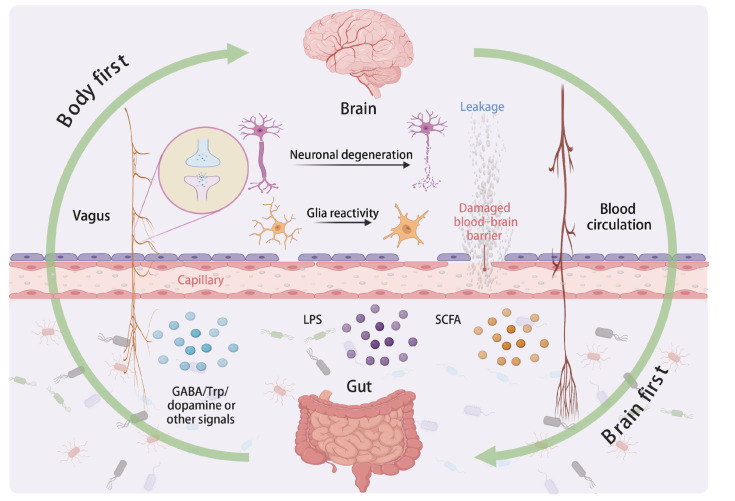
The microbiota–gut–brain axis enables bidirectional communication through interconnected neural, immune, metabolic, endocrine, and barrier-related pathways. The gut microbiota affects the development and functional regulation of the CNS through direct and indirect mechanisms that involve numerous microbial metabolites. Changes in the circulating levels of microbial products, such as LPS and SCFAs, along with any impairment of the gut and blood-brain barriers, all work together to regulate the translocation of neuroactive metabolites and pro-inflammatory mediators. It is worth mentioning that certain neuroactive microbial metabolites can get to central neuronal circuits through vagus nerve-mediated pathways. These various interactions lead to pathological processes, which are characterized by microglial hyperactivation and subsequent neurodegeneration. Created with BioRender.com. CNS: Central nervous system; GABA: gamma-aminobutyric acid; LPS: lipopolysaccharide; SCFA: short-chain fatty acids; Trp: tryptophan.

Over the past two decades, research has increasingly emphasized the critical role of microbiome in maintaining health and its link to neurodegeneration through the microbe-gut-brain axis. The intestinal epithelial permeability increases with age (Tran and Greenwood-Van Meerveld, 2013), allowing gut microbiota and its metabolites to cross the intestinal barrier into the somatic circulation, including the CNS. The gut microbiota communicates with the CNS through producing neuroactive substances, metabolites, and hormones that regulate the immune system, vagus nerve, enteric nervous system, neuroendocrine system, and circulatory system, forming a bidirectional regulatory network with the brain (El Aidy et al., 2015; Bistoletti et al., 2020; Silva et al., 2020). Based on differences in pathology propagation, two disease subtypes have been proposed: brain-first (**Figure 1**), where pathology originates in the CNS and extends to the peripheral autonomic nervous system, and body-first (**Figure 1**), in which pathology begins in the gut or peripheral autonomic nervous system and progresses to the CNS (Arotcarena et al., 2020; Leclair-Visonneau et al., 2020).

The gut microbiota and the host maintain a delicate balance, which is crucial for good health. Disruptions in this balance, known as dysbiosis, are strongly associated with the development of neurological disorders (Carding et al., 2015; Zhang et al., 2015). Advances in high-throughput sequencing technologies have revolutionized gut microbiome research, enabling comprehensive analysis of microbial diversity and host-microbe interactions (Li et al., 2021). More and more evidence shows that disorders of the gut microbiota are related to the pathologic progression of neurodegenerative diseases. These diseases include Alzheimer’s disease (AD), amyotrophic lateral sclerosis (ALS), Parkinson’s disease (PD), Huntington’s disease (HD), multiple sclerosis (MS), and frontotemporal dementia (FTD) (Liu et al., 2024b).

Neurodegenerative diseases (NDDs) encompass a broad range of neurological disorders characterized by the progressive degeneration of the central or peripheral nervous systems. These conditions pose a significant global health burden. As age-related disorders, NDDs share key pathological mechanisms, such as protein misfolding and aggregation, which lead to synaptic dysfunction and extensive neural network degeneration. With a rapidly aging world population, it is predicted that NDDs will become the second leading cause of death globally, following cardiovascular disease. The multifactorial pathogenesis of NDDs includes prion-like spreading of misfolded protein assemblies along thinly myelinated autonomic pathways, age-related declines in protein quality control that predispose individuals to amyloid formation, and impaired BBB integrity that limits therapeutic access (Mather, 2025). These molecular disorders manifest as progressive cognitive impairment, motor dysfunction, autonomic nervous system disorders, and neuropsychiatric symptoms in clinical practice. Currently, treatments primarily provide symptom relief rather than intervening to improve the disease itself. This situation underscores the need to deepen our understanding of the underlying mechanisms to formulate targeted neuroprotection strategies. In this context, intervention measures aimed at regulating the gut microbiota represent a promising approach, offering potential benefits in both experimental research and the clinical management of neurodegenerative diseases. This review addresses the fundamental role of the gut microbiota in both the direct and indirect regulation of neurological function. Specifically, the manuscript is organized into three key sections: (1) the significant effect of microbiota on the host through the regulation of age-related processes, vagus nerve activity, and intestinal barrier integrity; (2) the mechanisms of microbe-mediated neuroinflammatory processes, including cytokine production, immune system regulation, microglial activation, and pro-inflammatory responses; and (3) the relationship between intestinal microbiota ecology and the pathogenesis of neurodegenerative diseases, including clinical applications and associated risks. This review systematically clarifies the molecular pathways through which gut microbiota influence neural homeostasis and explores the underlying pathophysiological mechanisms linking microbial dysbiosis to neurodegenerative progression in the elderly.

## Search Strategy

**Figure 2:**
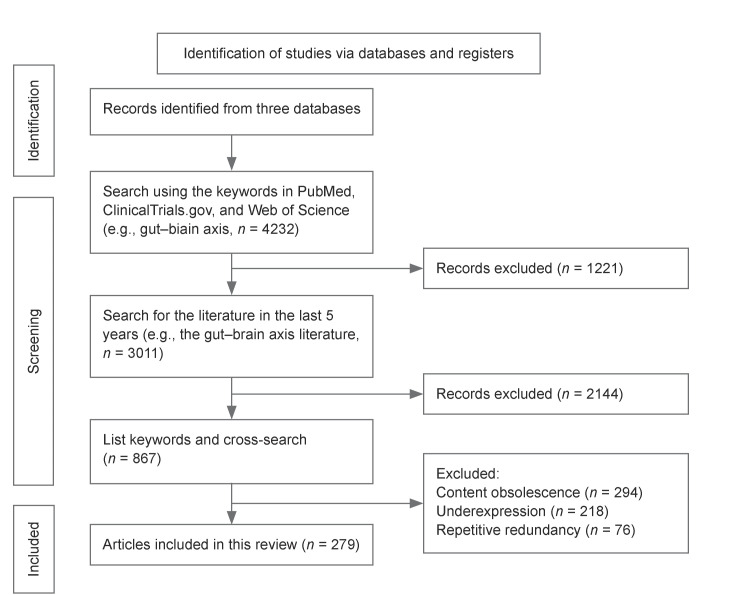
Flow diagram of the study selection procedure.

We conducted a comprehensive literature search in PubMed, ClinicalTrials.gov, and Web of Science from January to March 2025, using a systematic search strategy (**Figure 2**). Our search combined terms related to: (1) the microbiota-gut-brain axis (e.g., “gut–brain axis,” “intestinal barrier,” “vagus nerve,” “gut microbiota,” “microbiota-derived metabolites”); (2) NDDs (e.g., “Alzheimer’s disease,” “Parkinson’s disease,” “Huntington’s disease,” “amyotrophic lateral sclerosis,” “multiple sclerosis,” “frontotemporal dementia”); and (3) pathological mechanisms (e.g., “microglia,” “astrocytes,” “neuroinflammation,” “blood–brain barrier,” “protein aggregation,” “tau protein,” “α-synuclein”). The search used controlled vocabulary (MeSH terms) along with free-text keywords, organized into three conceptual domains: disease terminology, pathological mechanisms, and research methodologies. We reviewed titles and abstracts to identify clinical trials and mechanistic studies investigating how aging, gut barrier functions, and microbial metabolites influence CNS health through neuroimmune interactions. To capture relevant interventional research, we applied filters for clinical studies (“clinical trial” [Publication Type]) in both the PubMed and ClinicalTrials.gov databases. A total of 279 references were included in this review article.

## Timeline and Milestones of Research Development on the Microbiota–Gut–Brain Axis and Neurodegenerative Diseases

The development of microbiology is closely linked to ongoing innovations in sequencing technology, which have collectively enhanced our understanding of the microscopic world of life. In the 17^th^ century, Antonie van Leeuwenhoek first observed bacteria using a microscope he built himself, laying the foundational groundwork for microbiology. At the same time, humanity’s intuitive understanding of the connections between digestion, emotions, and behavior helped pave the way for the later development of the gut–brain axis theory (Miller, 2014). In 1959, Julian Pleasants from the University of Notre Dame successfully bred the first germ-free mouse, providing a vital model for in-depth research into the interactions between intestinal microbiota and their host (Pleasants, 1959). In 1998, Michael Gershon formally introduced the concept of the gut as the body’s “second brain” in his book, emphasizing the independence of the gut’s nervous system. This idea established a cognitive foundation for the gut–brain axis theory (Gershon, 1999) and encouraged the scientific community to reassess the intriguing connection between the gut and the brain. In 2002, seven laboratories from the European Union collaborated on the FAIR CT97-3035 project to develop molecular analysis methods for the human gut microbiota. They created a 16S rRNA sequence database from fecal samples, discovered that microbial diversity increases with age, designed phylogenetic probes, and validated the effectiveness of their methods in research (Blaut et al., 2002).

**Figure 3:**
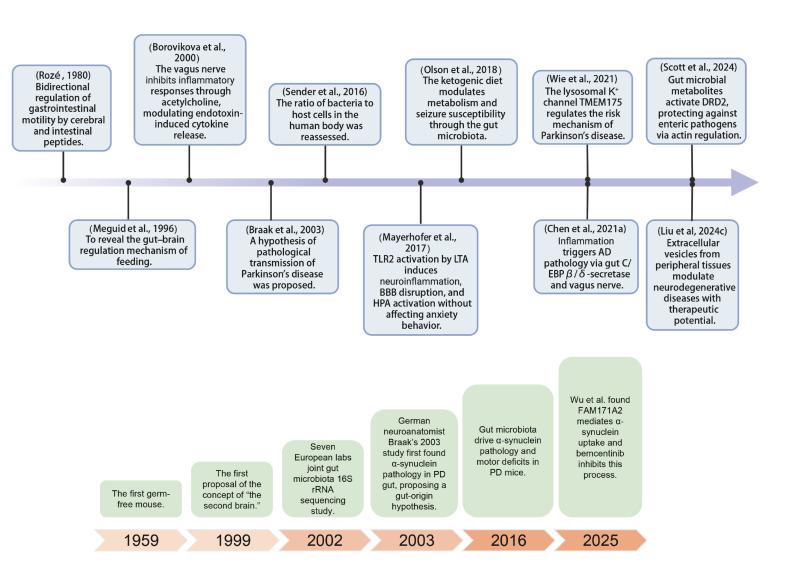
Timeline and milestones of research development on the microbiota–gut–brain axis and neurodegenerative diseases. AD: Alzheimer’s disease; BBB: blood–brain barrier; C/EBPbeta/delta: CCAAT/enhancer-binding protein beta/delta; DRD2: dopamine receptor D2; FAM171A2: family with sequence similarity 171 member A2; HPA: hypothalamic-pituitary-adrenal axis; LTA: lipoteichoic acid; TLR2: Toll-like receptor 2; TMEM175: transmembrane protein 175.

In the field of NDD research, in 2003, Braak et al. proposed the groundbreaking gut-origin hypothesis of PD through autopsy studies (**Figure 3**). He found that pathological aggregates of α-synuclein (α-syn) in the intestinal nervous system of patients with PD appeared earlier than in the midbrain region, and first suggested the possibility that pathological signals might be transmitted from the gut to the brain via the vagus nerve (Braak et al., 2003). In 2016, Sampson et al. conducted in-depth studies in mouse models overexpressing α-syn. The results showed that the gut microbiota can drive motor dysfunction, microglia activation, and pathological aggregation of α-syn. Further experiments revealed that antibiotic-induced gut microbiota clearance significantly improved these pathological phenotypes, while microbiota reconstitution exacerbated disease progression. These findings not only suggest that postpartum gut–brain signaling is involved in disease regulation but also advance research on the association between gut microbiota and NDDs to a new level. Recent studies have focused on the neuronal uptake mechanism of pathological α-syn fibers in PD, revealing that the risk gene FAM171A2 binds specifically to the C-terminal region of α-syn fibers via its extracellular domain 1 through electrostatic interactions (with a selectivity over 1000 times higher than monomers), thereby promoting fiber endocytosis and exacerbating neurotoxicity. Knocking down this gene, however, provides neuroprotective effects (Wu et al., 2025). More importantly, the compound bemcentinib has been shown to block this interaction *in vitro*, in cell models, and mice. This finding not only confirms the key role of FAM171A2 as an α-syn fibril uptake receptor but also provides a novel therapeutic target for PD.

In the evolution of microbial detection technology, Frederick Sanger’s invention of Sanger sequencing in 1977 marked the beginning of the first generation of sequencing technology. This technique is based on the principle of terminating DNA chain elongation using double-deoxyribonucleotide primers, and determines nucleotide sequences by electrophoresis to separate DNA fragments of different lengths. This technology laid a solid foundation for major research projects such as the Human Genome Project. However, as research demands grew, the first-generation sequencing technology was gradually replaced by second-generation sequencing technology due to its limitations including low throughput and high cost. Represented by the Illumina sequencing platform, second-generation high-throughput sequencing technology is based on the principle of sequencing by synthesis. It fixes DNA fragments onto a chip for bridge polymerase chain reaction amplification to form DNA clusters, and uses fluorescently labeled dNTPs for cyclic synthesis reactions. In each round of the reaction, laser detection of fluorescent signals determines the type of base. A single run can generate terabytes of data. Leveraging its advantages of high throughput and low cost, this technology is widely applied in fields such as microbial genome sequencing, transcriptomics analysis, and metagenomics research, making it one of the most widely used sequencing platforms in current research and clinical testing. In a study of patients with MS, quantitative analysis of microorganisms in patient stool samples was performed using multiple platforms, including Illumina MiSeq, Roche 454, microarray, and fluorescence in situ hybridization to perform quantitative analysis of the microbiota in patient fecal samples (Mirza et al., 2020). The results revealed that the relative abundance of *Methanobrevibacter* and *Akkermansiamuciniphilawas* significantly elevated in patients with MS. Meanwhile, Li et al. (2022) performed a meta-analysis of various neurodegenerative diseases using 16S rRNA gene amplification sequencing technology, which revealed an intrinsic association between gut microbiota α diversity and neurological disorders.

In the 21^st^ century, third-generation sequencing technology (single-molecule sequencing) emerged, with its core advantage being the ability to sequence single-molecule DNA directly without PCR amplification. Representative technologies include PacBio’s SMRT (detected via fluorescent signals) and nanopore sequencing (identified via changes in electrical current), with read lengths ranging from tens of kilobases to 1 megabase. This technology can span complex sequences in the genome and detect epigenetic modifications and other information. Although the single-base error rate is relatively high, errors can be corrected through multiple sequencing rounds and bioinformatics algorithms. These technologies are primarily applied in scenarios such as genome assembly, metagenomic analysis of complex communities, and full-length transcript sequencing, complementing second-generation sequencing technologies. Zhang et al. (2021) used 16S rRNA PacBio SMRT full-length gene sequencing technology in a study of a mouse model of recurrent stress-induced diarrhea to systematically investigate the differences in intestinal contents and mucosal microbiota and their association with serum 5-hydroxytryptamine (5-HT) concentrations, providing a new technical approach to elucidating the relationship between gut microbiota and intestinal dysfunction.

Looking back at the historical development of microbiology research, from early microscopic observations to the establishment of germ-free animal models, from the classification theory of the domains of life to the introduction of the gut–brain axis concept, each theoretical breakthrough has been driven by technological innovation; from Sanger sequencing to the iterative development of second- and third-generation sequencing technologies, microbial sequencing techniques have evolved from single-sequence determination into a powerful tool encompassing microbial genome sequencing and functional analysis, community structure and diversity analysis, evolutionary and phylogenetic studies, as well as pathogen detection and disease diagnosis. While we recognize the significant achievements in microbiology, we must also address the challenges that persist. Traditional selective plate culture methods tend to underestimate the abundance of *Bifidobacteria*, particularly *Bifidobacterium adolescentis*, and there are notable discrepancies in selectivity and recovery rates across various culture media. Furthermore, molecular biology techniques, despite their clear advantages, face limitations such as DNA degradation and variability in rRNA gene copy numbers (Apajalahti et al., 2003). A critical challenge lies in accurately identifying the types of microorganisms present and effectively translating foundational research on gut microbiota into practical *in vitro* clinical treatment protocols. Addressing these scientific hurdles is essential for advancing our understanding of microbiota–host interactions. Looking ahead, the integration of multi-omics technologies, decreasing sequencing costs, and the emergence of portable real-time detection methods promise to propel microbiology research forward. These advancements are expected to lead to significant breakthroughs in elucidating the mechanisms of microbiota-host interactions and in advancing precision medicine, ultimately offering new avenues for improving human health and enhancing disease prevention and control.

## Enteric Microorganisms

### Role of microorganisms in the host

The human gastrointestinal tract harbors trillions of microorganisms that constitute the gut microbiota, a dynamically balanced ecosystem critical for maintaining overall host health (Loh et al., 2024). This microbiota supports health by facilitating nutrient absorption and synthesizing essential metabolites, including lipids, amino acids, vitamins (such as vitamin K and B vitamins) (Uebanso et al., 2020), and short-chain fatty acids (SCFAs) (LeBlanc et al., 2013; Hou et al., 2022). SCFAs, such as acetate, propionate, and butyrate, play a crucial role in mediating gut–microbiota interactions, regulating immune responses, inflammatory pathways, and maintaining epithelial integrity (Cummings et al., 1987; Dalile et al., 2019).

However, with aging, the gut microbiota becomes increasingly prone to dysbiosis. This condition is characterized by elevated levels of lipopolysaccharides (LPS) derived from Gram-negative bacteria, which can trigger chronic inflammation (Ma et al., 2025). Concurrently, the reduction in SCFA production leads to inadequate mucus production and increased intestinal barrier permeability. These changes exacerbate systemic low-grade inflammation and contribute to the pathogenesis of aging-related diseases (Thevaranjan et al., 2017; Silva et al., 2020).

A stable gut microbiota supports host health through the synthesis of neurotransmitters, such as glutamate, γ-aminobutyric acid (GABA), dopamine, and serotonin (5-HT). The vagus nerve plays a key role in transmitting these signals (Clarke et al., 2014; Chen et al., 2021b; Socala et al., 2021). Current interventions targeting the gut microbiota, including fecal microbiota transplantation (FMT), prebiotics, probiotics, and synbiotics, have shown promising results in modulating gut health (Kassam et al., 2013; Pandey et al., 2015; Ansari et al., 2020; Barbosa and Vieira-Coelho, 2020). Moreover, recent studies highlight the methanogenic archaeon *Methanobrevibacter smithii* as a key modulator of cognitive functions. This microorganism can remodel microbiota composition, regulate energy metabolism, and influence metabolic pathways involving butyrate, histidine, linoleic acid, and phenylalanine to enhance cognition (Fumagalli et al., 2025). Research using a mouse FMT model supports these mechanisms and opens new therapeutic avenues for microbiota-based interventions in cognitive impairment.

### Effects of aging on gut microbiota

The initial colonization of the gut microbiota originates mainly from maternal skin and vaginal bacteria. Adult intestinal microbiota gradually takes the place of these early microbial communities. During the critical developmental window in early life, this microbial ecosystem exhibits limited diversity and is dominated by *Bifidobacteria* (Romano-Keeler and Sun, 2022; Nunez et al., 2025). The changes in the dietary structure during infancy can lead to a big restructuring of the gut microbial community, which is also notably affected by environmental factors (Agosti et al., 2017).

The gut microbiota has a high level of plasticity during this crucial period. This makes it more likely to be affected by adverse environmental exposures. It can disrupt the finely tuned microbial succession and might have an effect on developmental health by causing dysbiosis (Ville et al., 2020). This adaptation to the environment, which is brought about by the dynamic changes in bacterial composition and metabolic functions, is under strict control by the host’s immune system. Notably, microbial dysbiosis poses health risks beyond developmental stages: analogous mechanisms of physiological imbalance reemerge during aging. As a complex, time-dependent process featuring progressive declines in physiological, immune, metabolic, and genomic functions, aging is accompanied by significant shifts in microbial diversity (O’Toole and Jeffery, 2015; Wilmanski et al., 2021).

In younger mice, *Parabacteroides* and *Akkermansia* levels are higher, whereas these bacteria are significantly reduced in older mice. *Akkermansia* promotes intestinal mucus secretion, which helps restore and maintain intestinal barrier integrity (Rodrigues et al., 2022). Reaearch in both human and animal models has shown that aging is associated with increased intestinal permeability and elevated colonic cytokine expression, contributing to chronic systemic inflammation, a hallmark of aging (Thevaranjan et al., 2017). Research has demonstrated that transplanting gut microbiota from aged mice into young germ-free mice leads to a decline in *Akkermansia* and an increase in TM7 bacteria and *Proteobacteria*. This shift results in small intestine inflammation, bacterial translocation into the bloodstream, and systemic T-cell activation, indicating that aged microbiota can promote inflammatory aging in young hosts (Fransen et al., 2017). However, oral administration of *Akkermansia* can mitigate aging-related intestinal phenotypes in aged mice (Shin et al., 2021). 16S rRNA sequencing analysis has revealed that the abundance of specific bacterial families, including *Ruminococcaceae*, *Clostridiaceae*, and *Enterobacteriaceae*, is age-dependent in the fecal microbiota of young and aged mice (Conley et al., 2016). Aged mice exhibit elevated serum monocyte chemoattractant protein-1 levels, indicating an inflammatory phenotype. The abundance of certain microbes correlates with monocyte chemoattractant protein-1 levels, suggesting potential interactions with the immune system (Conley et al., 2016).

Aging also alters microbial-controlled metabolic pathways, such as GABA biosynthesis and SCFA production, with these metabolite levels lower in aged mice compared with younger mice. Specifically, *Proteobacteria* and *Enterobacteriaceae* increase, while *Bifidobacterium* and *Clostridium* decrease (Xu et al., 2019; Xiao et al., 2025), leading to reduced production of SCFAs. Additionally, aging is accompanied by changes in autophagic flux, where the impaired clearance of proteins leads to their accumulation (Ghavami et al., 2014). The mammalian target of rapamycin (mTOR) signaling pathway is negatively correlated with autophagic flux, promoted by *Clostridium butyricum* and inhibited by *Lactobacillus* X12 in healthy individuals (Gao and Tian, 2023).

## Vagus Nerve Regulation and Intestinal Barrier Integrity

### Vagus nerve

The vagus nerve, a key component of the parasympathetic nervous system, operates in conjunction with the sympathetic nervous system to form the autonomic nervous system, providing a direct link between the brain and the enteric nervous system (Bonaz et al., 2018). As one of the body’s most widely distributed and complex nerves (Han et al., 2022), the vagus nerve facilitates information exchange between the brain and various organs (such as ears, heart, lungs, pancreas, spleen, and gastrointestinal tract). Its regulatory role in brain-body communication arises in the nucleus tractus solitarius within the brainstem and involves the immune system.

The enteric nervous system is a component of the peripheral nervous system and serves as a key interface connecting the host and gut microbiota. It processes sensory signals from the autonomic nervous system to control gastrointestinal motility and secretory functions. These include regulating peristalsis, digestive enzyme secretion, and the release of hormones such as gastrin and secretin (Browning and Travagli, 2014). Bottom-up signaling from the gut reaches the CNS via the autonomic nervous system. In response, the CNS sends top-down feedback to the gut, establishing a bidirectional communication loop (Durgan et al., 2019). The vagus nerve-mediated gut–brain axis is crucial for maintaining physiological balance. It plays a central role in the development and progression of psychiatric, neurological, and inflammatory disorders.

A groundbreaking study showed that vagus nerve stimulation notably reduced the release of pro-inflammatory cytokines, such as tumor necrosis factor-α (TNF-α) and interleukin (IL)-1β (IL-1β), in a lipopolysaccharide-induced sepsis model (Borovikova et al., 2000). This finding prompted researchers to conceptualize the “cholinergic anti-inflammatory pathway,” identifying the vagus nerve as a physiological regulator that acts as a neural “brake” on inflammatory responses (Tracey, 2002). Stimulation of vagal afferent fibers by inflammation activates the hypothalamic-pituitary-adrenal axis. It also triggers neural pathways linked to sickness behavior. Certain gut pathogens can block the transmission of inflammatory IL-1β signals to the vagus nerve. By doing so, they prevent the anorexia induced by hypothalamic stimulation (Rao et al., 2017).

A recent study indicates that the gut microbiota modulates host locomotor behavior through glucagon-like peptide-1 (GLP-1) and vagal signaling. In mice, broad-spectrum antibiotic treatment has been shown to reduce locomotion while elevating GLP-1 levels (Lai et al., 2024). Colonization with specific microbes (e.g., *Lactobacillus reuteri* and *Bacteroides thetaiotaomicron*) suppresses GLP-1 and reduces locomotor deficits, indicating that gut microbes regulate motor behavior through enteroendocrine and vagal-dependent pathways (Lai et al., 2024).

In fecal microbiota transplantation experiments, transplantation from chronic social defeat stress-susceptible mice induced anhedonia-like phenotypes in antibiotic-treated mice, accompanied by elevated plasma inflammatory markers. These phenotypes were strongly linked to colonization by *Lactobacillus intestinalis* and *Lactobacillus reuteri*. Subdiaphragmatic vagotomy completely blocked the microbiota-mediated behavioral abnormalities and neuroinflammatory responses (Wang et al., 2020).

The collective evidence highlights the vagus nerve as a key mediator in the interplay between gut microbiota, neuroendocrine signaling, and brain function, suggesting potential therapeutic targets for a range of neuropsychiatric and inflammatory conditions.

### Intestinal barrier

The gastrointestinal tract is a unique organ system where dynamic interactions occur between host cells and a complex environment, extending well beyond basic food digestion. The intestinal barrier, comprising the mucus layer, epithelial barrier, and gut–vascular barrier, collectively provides robust protection against external threats. However, in various NDDs, compromised intestinal barrier function can lead to microbial dysbiosis and pathological changes. Indeed, alterations in gut microbiota and intestinal barrier damage have been reported in patients with mild cognitive impairment, AD, PD, and ALS (Pellegrini et al., 2023).

Dietary fiber and its metabolites play key roles in maintaining intestinal barrier function and microbial homeostasis. A high fiber intake increases the production of dietary fiber metabolites and SCFAs, which stimulate mucus secretion to sustain a robust and intact mucus layer. Importantly, dietary fiber also enhances the resilience of human gut microbiota to damage by limiting microbial overgrowth and maintaining a balanced interspecies network (Makki et al., 2018). Sodium butyrate (from dietary fiber fermentation) activates AMP protein kinases to promote tight junction reorganization, protecting gut barrier function (Miao et al., 2016).

With aging, gradual deterioration of the intestinal barrier permits pro-inflammatory gut microbial metabolites and pathogenic bacterial products to enter the bloodstream, exacerbating systemic inflammatory responses and progressively compromising BBB integrity, ultimately leading to CNS inflammation and neurodegeneration (Pellegrini et al., 2023). In constipated mice, gut dysbiosis has been shown to induce inflammation and enhance permeability of both the intestinal barrier and BBB, altering the expression of regulatory T cells (Tregs) and helper T cells in the nervous system, adversely affecting autoimmune encephalomyelitis (Lin et al., 2021). Moreover, elevated glial markers (such as glial fibrillary acidic protein, and Sox-10) in the gastrointestinal tract of patients with PD are linked to pro-inflammatory cytokines (Natale et al., 2021).

As a fundamental component of the adaptive immune system, Th17 cells play a significant role in defending against opportunistic pathogens of fungal or bacterial origin, particularly in intestinal barrier protection, where they regulate the host’s response to the microbiota and maintain intestinal homeostasis (Stockinger and Omenetti, 2017). Th17 cells are highly pro-inflammatory, and the characteristic cytokine IL-17 they secrete promotes the expression of multiple cytokines by activating nuclear factor κB (NF-κB) (Witowski et al., 2004). This cell population and IL-17, due to their cytokine profile and ability to recruit other immune cells, are involved in the induction of various autoimmune diseases, such as multiple sclerosis, inflammatory bowel disease (IBD), rheumatoid arthritis, and psoriasis (Schinocca et al., 2021).

Type 17 immunity, a specialized branch of the immune system, is primarily mediated by group 3 innate lymphoid cells and Th17 cells, among other innate and T lymphocyte subsets. Upon regulation by IL-23, these cells can act on intestinal epithelial cells by secreting IL-17A/F and IL-22, thereby increasing mucus, antimicrobial peptides, chemokines, and tight junction proteins, thereby promoting wound healing and enhancing intestinal barrier integrity (Blaschitz and Raffatellu, 2010; Keir et al., 2020; Ohara et al., 2024). Regen et al. (2021) found that IL-17-deficient mice exhibited reduced susceptibility to experimental autoimmune encephalomyelitis in a multiple sclerosis animal model, but this susceptibility returned to normal after restoration of IL-17 expression in the intestinal microbiota or intestinal epithelium. Another study showed that infection with enterotoxin-producing *Escherichia coli* can induce intestinal IL-17 expression through GABA-producing gut microbiota, as antibiotic treatment reduces IL-17 levels, while GABA supplementation restores its expression via the mTORC1 signaling pathway, highlighting the role of GABA in IL-17-mediated intestinal immunity (Ren et al., 2017). A meta-analysis showed that serum IL-17 levels were significantly higher in patients with AD and ALS compared with controls. In contrast, no significant differences were observed in patients with PD or MS, suggesting that IL-17 may play a role in regulating neuronal inflammation and could serve as a therapeutic target for specific NDDs (Gautam et al., 2023). Another study showed that propionic acid levels were elevated in male AD mice following antibiotic treatment, and this was achieved by reducing the secretion of peripheral Th17 cells and IL-17, thereby decreasing reactive astrocyte proliferation and amyloid-β plaque deposition; RNA sequencing of astrocytes revealed reduced expression of pro-inflammatory genes and increased expression of neurotrophic genes. These findings suggest that propionic acid or strategies to promote its production may target the gut–brain axis via IL-17-dependent pathways, offering a novel approach for treating AD (Chandra et al., 2025).

The corresponding ligand for IL-17, IL-17R, is widely distributed in various tissues and the CNS. Monomeric IL-17A and IL-17F signal through the formation of homodimers and heterodimers, respectively, and bind to specialized dimeric receptor complexes, IL-17RA and IL-17RC (Walliser and Göbel, 2018). A study has shown that levels of IL-17A/IL-17RA in the human CNS increase with age and are significantly elevated in patients with PD. Activation of neuronal IL-17A has been observed in the brains of patients with PD and in α-syn mouse models, where recombinant IL-17A exacerbates α-syn pathological deposits. Blocking the IL-17A/IL-17RA pathway via antibodies or gene knockdown alleviates the effects of α-syn, suggesting that modulating this pathway may disrupt the harmful cycle driving PD progression (Yan et al., 2025).

Intestinal tight junction (TJ) proteins, including occludin, claudins, and zonula occludens (ZOs), are crucial for preserving epithelial barrier integrity (Chelakkot et al., 2018). A primary function of intestinal epithelial cells is to maintain this barrier while allowing the passage of essential ions, nutrients, and water, and simultaneously preventing bacterial toxins and pathogens from entering (Rescigno, 2011). Acting as “gates and fences,” TJ proteins enable the paracellular transport of certain molecules and block the transmembrane passage of proteins, lipids, and microbe-derived peptides (Berkes et al., 2003). Any structural alterations to TJs can be detrimental to the organism.

The intestinal epithelial barrier relies on an intricate network of TJ complexes composed of transmembrane proteins (such asoccludin, claudin family members, and tricellulin) and cytoplasmic scaffolding proteins (e.g., ZO-1/-2/-3, cingulin, junctional adhesion molecules). TNF-α, interferon-γ (IFN-γ), and IL play key roles in regulating the completion (Capaldo and Nusrat, 2009). Marchiando et al. (2010) found that TNF-α induces the endocytosis of occluding proteins through the caveolin-1-dependent pathway, damaging barrier function. The increased level of occludin can alleviate this kind of cytokine-mediated damage (Marchiando et al., 2010). Furthermore, the activation of the NF-κB signaling pathway by TNF-α is another important regulatory mechanism. To protect the mice from severe dehydration and diarrhea, the inhibition of NF-κB helps maintain the barrier characteristics of intestinal epithelial cells.

Research reveals IL-1β enhances Caco-2 monolayer permeability through NF-κB activation without inducing apoptosis (Al-Sadi and Ma, 2007). A previous study has shown that physiological concentrations of IL-1β are capable of undermining the TJ barrier in a manner that is both concentration-dependent and time-dependent, thus furnishing some understanding regarding barrier dysfunction during intestinal inflammation (Al-Sadi and Ma, 2007). Meanwhile, IFN-γ, which is primarily associated with inflammatory immune responses, has elevated levels in the intestinal mucosa with IBD (Stallmach et al., 2003). IFN-γ further augments barrier permeability by reducing the expression of ZO-1 and occludin through the activation of AMP-activated protein kinase, a mechanism that is independent of cellular energy levels (Scharl et al., 2009).

Besides cytokines, intestinal permeability also depends on ion channels, which are crucial for maintaining ionic homeostasis and responding to mechanical and chemical stimuli. Ion balance regulates cell secretion, absorption, signaling transduction, and mechanical stress. The piezoelectric channel exists in large quantities in the intestinal tract and regulates many physiological and pathological processes, making it a promising therapeutic target (He et al., 2024). Human 5-HT-producing enterochromaffin cells (Linan-Rico et al., 2016) rely on Piezo2 channels to detect mechanical stimuli and induce 5-HT release, regulating gut motility, secretion, and visceral sensation (Linan-Rico et al., 2016). A gut ssRNA modulates 5-HT synthesis through Piezo1 activation, affecting both intestinal peristalsis and bone formation, which implies that the ssRNA-Piezo1 axis could be a potential therapeutic avenue for skeletal and intestinal disorders (Sugisawa et al., 2020).

**Figure 4:**
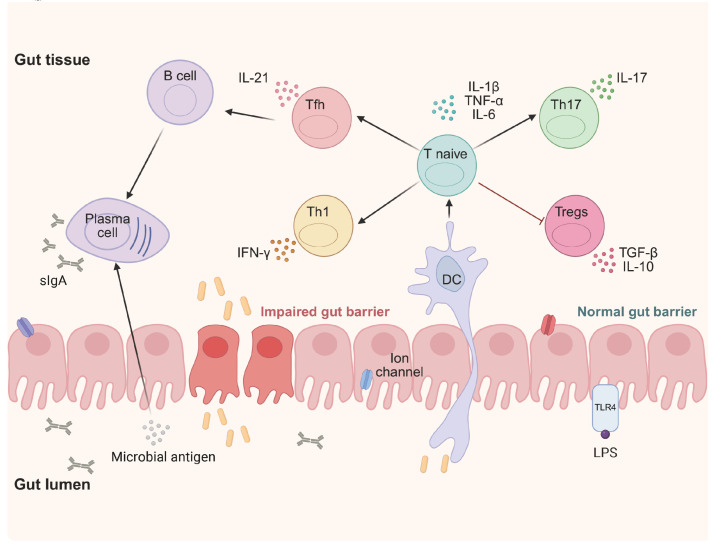
Impaired gut barrier triggers a series of pathological events. The ecological disturbance promotes the proliferation of harmful bacteria, leading to the production of toxic metabolites (such as LPS), activation of the TLR4/NF-κB pathway, and the release of pro-inflammatory cytokines (such as TNF-α and IL-6). This condition increases intestinal permeability, allowing LPS and pathogens to enter the circulatory system. The heightened immune response exacerbates the inflammatory microenvironment and disrupts the balance of Th17/Treg cells. Meanwhile, the secretion of SigA decreases, further weakening mucosal immunity and worsening ecological imbalance and barrier dysfunction. Additionally, dysfunction of intestinal ion channels (such as TRPV4 and Piezo1) impairs ion homeostasis. Disrupted ionic balance hinders epithelial repair and the integrity of the gut barrier. This series of immune responses leads to metabolic disorders and neuroinflammation. Created with BioRender.com. IFN-γ: Interferon-gamma; IL-1β: interleukin-1 beta; IL-6: interleukin-6; IL-10: interleukin-10; IL-17: interleukin-17; IL-21: interleukin-21; LPS: lipopolysaccharide; SigA: secretory immunoglobulin A; TGF-β: transforming growth factor-beta; TLR4: Toll-like receptor 4; TNF-α: tumor necrosis factor-alpha.

Transient receptor potential vanilloid 4 (TRPV4) contributes to visceral hypersensitivity by stimulating ATP/glutamate release from mucosal cells, activating colonic afferents (**Figure 4**; Meng et al., 2025). Moreover, TRPV4-expressing muscularis macrophages regulate intestinal smooth muscle contraction through prostaglandin E2 release, maintaining normal gastrointestinal motility, and offering novel therapeutic targets for motility disorders (Luo et al., 2018). At the same time, the lysosomal ion channel TMEM175 has been associated with PD through mutations that lead to lysosomal voltage dysregulation and dysfunction (Wie et al., 2021; Tang et al., 2023). In TMEM175-deficient mice, the worsened intestinal inflammation and related nerve changes reveal new insights into how microbes and the nervous system closely interact (unpublished data). All of these findings show new elements of the communication pathways between organs. In particular, they shed light on the bidirectional mechanisms related to gastrointestinal homeostasis and the integrity of the CNS.

## Microbiota–Gut–Brain Axis

The microbiota–gut–brain axis is regulated in a complex manner by a diverse array of neurotransmitters, neuropeptides, and microbes-derived metabolites. The gut microbiota and the CNS communicate through two main mechanisms. This bidirectional communication involves both neural (vagal) and humoral pathways (Fröhlich et al., 2016; Fülling et al., 2019).

Neurotransmitters have diverse effects on gut ecology, and the microbiota is capable of directly synthesizing and releasing neurotransmitters. Gut microbiota dysbiosis can compromise the integrity of TJ proteins, leading to increased intestinal permeability, often referred to as “leaky gut.” The gastrointestinal tract contains the highest density of immune cells in the body and secretes cytokines and chemokines that enter the bloodstream and lymphatic system. This immune signaling has the potential to disrupt neural communication mediated by the vagus nerve and spinal afferent neurons, thereby influencing brain function (Jacobson et al., 2021).

The microbiota regulates the immune system through host-microbe interactions and metabolic pathways. Host-microbe interactions occur when cells in the host come into contact with bacterial membrane components within the intestinal mucosa. Additionally, metabolic activities of the intestinal microbiota generate a complex network of interactions, such as the production of SCFAs and the regulation of polyunsaturated fatty acid metabolism. These interactions exert indirect immunomodulatory effects in the peripheral and CNS and further emphasize the complicated interactions of the microbiota-gut-brain axis (Chen et al., 2022; Thakur et al., 2023).

### Neuroactive metabolites of microorganisms

The gut microbiota can produce, modulate, or consume various neurotransmitters, including 5-HT, dopamine, norepinephrine (NE), and GABA (Strandwitz, 2018). Additionally, neuropeptide synthesis is influenced by hormone and amino acid availability, and these factors are in turn regulated by the microbiota (Holzer, 2016). It should also be mentioned that neuropeptides can feed back to the gut microbiota, shaping its composition and function in a bidirectional manner (Holzer, 2016).

Microbial metabolism of amino acids such as tryptophan, tyrosine, and phenylalanine has an effect on the production of neurotransmitters, which include 5-HT, NE, and dopamine (DA) (Dodd et al., 2017). Tryptophan metabolism deserves special attention because its microbes-derived metabolites (such as indole and kynurenine) can alter glutamatergic signaling and are associated with neurobehavioral abnormalities (Schwarcz et al., 2012; Lukic et al., 2019). Kynurenine, a major tryptophan metabolite (Sheibani et al., 2023), plays an important role in NAD^+^ synthesis, inflammation linked to neurological disorders, and immune regulation. The research on animals shows that supplementing probiotics can increase plasma tryptophan levels, alleviate stress-induced neuroinflammation, and changes in central NE levels (Desbonnet et al., 2008, 2010).

DA is the biochemical precursor of NE, and both are derived from the amino acid tyrosine. Using mucosal analysis, a comprehensive study revealed distinct monoamine alterations in IBDs (Magro et al., 2002). Key findings include: (1) a decreased level of NE in the mucosa (both inflamed and non-inflamed regions) of patients with Crohn’s disease, while levels remain unchanged in patients with ulcerative colitis; (2) elevated levodopa (L-DOPA) and decreased DA in the inflamed mucosa of both diseases, reflecting impaired decarboxylase activity (as evidenced by a reduced DA/L-DOPA ratio); and (3) significant depletion of 5-HT in the inflamed mucosa compared with healthy mucosa. These disease-specific neurotransmitter changes suggest divergent pathophysiological mechanisms in Crohn’s disease and ulcerative colitis (Magro et al., 2002). Collectively, intestinal inflammatory processes may considerably affect the cellular mechanisms responsible for the synthesis and storage of DA, NE, and 5-HT, potentially contributing to the pathogenesis of IBDs. The dysregulation of monoamine metabolism in IBD raises important questions regarding the underlying molecular drivers of these alterations.

Insights from animal models further highlight the role of the gut microbiota in modulating neurotransmitter availability. Specifically, there is evidence that specific pathogen-free mice maintain high levels of free DA and NE in their intestinal lumen, whereas germ-free mice predominantly harbor these catecholamines (CA) in biologically inactive conjugated forms, leading to remarkably lower free CA levels (Asano et al., 2012). Notably, co-culturing germ-free mice with *Clostridium* species possessing β-glucuronidase activity or with fecal microbiota from specific pathogen-free mice substantially elevated free CA levels. In contrast, inoculation with β-glucuronidase-deficient *E. coli* mutants failed to produce this effect (Asano et al., 2012).

The gut microbiota also contributes to the production of excitatory (glutamate) and inhibitory (GABA) neurotransmitters. Dysregulation of glutamate/GABA signaling is implicated in conditions such as autism spectrum disorder, schizophrenia, and major depressive disorder (Bravo et al., 2011). While the role of GABA in the CNS is well understood, the mechanisms by which intestinal glutamate and GABA cross the BBB remain debated, potentially involving passive diffusion or transporter-mediated pathways (Bravo et al., 2011). Large-scale analyses of commercial probiotics show strain-specific GABA secretion by L*evilactobacillus brevis* and *Lactiplantibacillus plantarum*, both *in vitro* and *in vivo* (Monteagudo-Mera et al., 2022). Additionally, using CRISPR/Cas12a-mediated gene knockout to generate a mutant lacking the glutamate decarboxylase gene (gadB), the study revealed that GABA produced by *Bacteroides finegoldii* considerably modulates intestinal inflammation while exerting a limited effect on gut barrier integrity (Yousuf et al., 2025). These findings provide the first comprehensive evidence of the capacity of *Bacteroides* species to synthesize neuroactive metabolites and underscore their essential immunomodulatory functions.

GABA receptors regulate the release of monoamine neurotransmitters and hormones from endocrine cells, such as serotonin, which is integral to CNS functions including behavior, mood, sleep, and appetite. Furthermore, emerging evidence highlights the role of astrocytic Ca^2+^ signaling in major depressive disorder. Specifically, it influences the condition by regulating 5-HT pathways (González-Arias et al., 2023).

Moreover, the microbiota induces the secretion of neurotrophic factors that are vital for neuronal development and function, such as nerve growth factor, glial cell line-derived neurotrophic factor, and brain-derived neurotrophic factor (BDNF) (Maqsood and Stone, 2016). These neurotrophins are involved in the pathogenesis of NDDs. BDNF is essential for neuronal growth, survival, synaptic differentiation, and pathological processes in the CNS. Germ-free adolescent mice show reduced BDNF expression in the cortex and amygdala compared with controls (Desbonnet et al., 2015). Additionally, transgenic mice with hippocampal cognitive deficits show decreased BDNF expression at both protein and mRNA levels (Bonfili et al., 2021).

### Neuroinflammation and immune response

There is growing evidence that age-related changes in the composition of gut microbiota contribute to neuroinflammation, which is crucial in both aging and age-associated cognitive decline (Alsegiani and Shah, 2022). Compared with young mice, aged mice exhibit gut dysbiosis, elevated plasma and brain lipopolysaccharide levels, and activation of Toll-like receptor (TLR)/NF-κB signaling pathways, ultimately impairing cognitive function (Wu et al., 2021). Inflammatory signaling along the gut-brain axis operates bidirectionally, involving both afferent (“gut–to–brain”) and efferent (“brain–to–gut”) pathways, through which regulatory responses are elicited to restore homeostasis or exacerbate inflammation.

Microglia are the resident immune cells of the brain. One mechanism that affects the microbiota–gut–brain axis involves the maturation and function of microglia in the context of inflammation (Cryan and Dinan, 2015; Erny et al., 2015). Microglia not only regulate neuroinflammatory processes but also participate in synaptic plasticity, CNS maturation, and the clearance of debris and aggregates (Colonna and Butovsky, 2017). A previous study reveals that reactive and senescent astrocytes accumulate with age in NDDs, triggering neuroinflammatory responses that adversely affect the surrounding tissue microenvironment. This persistent inflammatory state and alteration of the microenvironment are key drivers of cognitive decline in the elderly (Cohen and Torres, 2019).

It is a well-known fact that the gastrointestinal tract interacts with the CNS. Additionally, the interplay between gut microbiota and the host immune system collectively regulates the production and release of neurotransmitters, neuropeptides, cytokines, and other signaling mediators. Compared with younger populations, centenarians display increased plasma concentrations of proinflammatory cytokines such as TNF-α, IL-8, and IL-6 (Wu et al., 2022). Interferons, which are inflammatory cytokines produced by Th1 cells (**Figure 4**), function in the hippocampus to reduce GABAergic inhibition, leading to network hyperexcitability and altering social behaviors (Zhu et al., 2011). Th17 cells are powerful inflammatory effector cells that differentiate within the lamina propria of the small intestine under the influence of certain gut commensal bacteria, especially *Cytophaga-Flavobacter-Bacteroidetes* (Ivanov et al., 2008). In the absence of Th17-inducing microbiota, Tregs become more prevalent in the lamina propria. Thus, gut microbiota modulates the Th17/Treg equilibrium, shaping intestinal immunity, tolerance, and vulnerability to IBDs (Ivanov et al., 2008).

Microglial activation and function can be triggered by various factors arising from host-bacterial interactions (Huang et al., 2023). These factors include cytokines, tryptophan metabolites, bacterial membrane components (e.g., peptidoglycans and LPS) (Yamawaki et al., 2018), and bacterial metabolites (such as SCFAs). Signals that originate from the gut can reach the brain via the bloodstream, the vagus nerve (Zhou et al., 2022), or the meningeal lymphatic system, affecting γδ T cells (Park et al., 2022).

In PD models, α-syn suppresses microglial autophagy by activating TLR4-dependent p38 mitogen-activated protein kinase (MAPK) phosphorylation and upregulating the AKT-mTOR signaling cascade (Tu et al., 2021). Microglial phagocytic activity is critical for clearing Aβ (Podlesny-Drabiniok et al., 2020), tau proteins (Odfalk et al., 2022), and α-syn. However, in both aging and NDDs, microglial phagocytic dysfunction allows for the gradual accumulation of toxic molecules, contributing to cognitive decline (Podlesny-Drabiniok et al., 2020). SCFAs are also helpful in diminishing oxidative damage from intracellular lipid peroxidation; for instance, acetate modulates immune responses by influencing the c-Jun N-terminal kinase (JNK), MAPK, and NF-κB pathways. Apart from SCFAs, elevated systemic levels of LPS are associated with microglial activation, neuronal death, cognitive deficits, and cytokine-driven sickness behaviors (such as reduced locomotion, decreased food intake, and social withdrawal; Zhao et al., 2019). During the perinatal period, the migration of CD4^+^ T cells to the brain is important for microglial maturation, a process potentially governed by gut microbiota (Pasciuto et al., 2020). With aging, the numbers of monocytes, CD4^+^, and CD8^+^ T cells rise, while other cell subsets, such as dendritic cells and B cells, tend to be underrepresented (Mogilenko et al., 2022).

Several neurological disorders are associated with an increase in intestinal permeability. The gut contains the body’s largest contingent of immune cells, which constantly monitor the intestinal environment. Dysbiosis is often accompanied by a loss of microbial diversity and an increase in proinflammatory microbes, leading to the upregulation of inflammatory markers in the CNS. Furthermore, dysbiosis can damage the intestinal epithelium, exposing gut and immune cells to bacterial elements and exogenous metabolites. Along with a disrupted CNS barrier, this series of events enables gut-derived molecules, toxins, and pathogens to penetrate the brain parenchyma, activating local immune cells and inciting neuroinflammation (Seguella and Gulbransen, 2021). Intestinal antigens prompt B cells to differentiate into immunoglobulin A (IgA)-secreting plasma cells, modulating the gut microbial community. In autoimmune disorders of the nervous system, a large number of IgA-positive plasma cells migrate to the brain and spinal cord, alleviating neuroinflammation in an IL-10-dependent manner (Rojas et al., 2019). These findings provide the opportunity to develop new therapies for neuroimmune diseases using gut antigens to increase the production of IgA-positive B cells and enhance the suppression of neuroimmune responses (Rojas et al., 2019; Fitzpatrick et al., 2020).

### Blood–brain barrier

The permeability of the BBB changes, and the infiltration of peripheral immune cells play an important role in activating neuroglial cells, which is a key feature of neuroinflammation. This activation triggers the secretion of inflammatory mediators, resulting in neurotoxic effects and impaired neuronal function (Chitnis and Weiner, 2017). Furthermore, the activation of glial cells can destabilize the BBB, perpetuating a cycle that exacerbates the neuroinflammatory cascade (Takata et al., 2021). The BBB is a sophisticated multicellular interface, composed of brain endothelial cells, TJs, and adherens junctions (AJs), which are essential for maintaining CNS homeostasis. Unlike peripheral endothelial cells, brain endothelial cells lack fenestrations and restrict paracellular diffusion via TJs and AJs. TJs are formed by transmembrane proteins (such as occludins and claudin-1/3/4/5/12) and cytoplasmic scaffolding proteins (ZO-1/2/3), while AJs strengthen intercellular adhesion through cadherins, catenins, PECAM-1, and junctional adhesion molecules (Sasson et al., 2021; Shashikanth et al., 2022). Notably, ZO-1 anchors claudins and occludins to the cytoskeleton, and these proteins play a key role in maintaining BBB structural integrity and functional permeability.

The BBB selectively regulates molecular transport, which allows crucial nutrients such as SCFAs (including butyrate, propionate, and acetate) to cross via monocarboxylate transporters. Besides serving as energy substrates for colonic epithelia, SCFAs modulate systemic immunity through gene regulation and directly influence BBB integrity and neuroglial function in neurological disorders (O’Riordan et al., 2022). For example, Caetano-Silva et al. (2023) demonstrated that germ-free mice exhibit impaired microglial maturation, which can be reversed by colonization with SCFA-producing microbes. Using *in vitro* BBB models, it has been shown that butyrate and propionate reorganize actin cytoskeletal dynamics, stabilize TJ protein localization, counteract LPS-induced barrier disruption, and regulate mitochondrial networks. These findings suggest the dual role of SCFAs in maintaining and repairing the BBB (Fock and Parnova, 2023).

Specific pathogen-free mice exhibit reduced expression of claudin-5 and occludin in the hippocampus. Meanwhile, treatment with probiotics can restore the levels of TJ proteins and the integrity of the barrier (Braniste et al., 2014). Similarly, germ-free mice demonstrate increased BBB permeability and TJ impairments. These phenotypes can be reversed when the mice are colonized with bacteria that produce SCFAs (Braniste et al., 2014). Fecal microbiota transplantation partially rescues antibiotic-induced BBB dysfunction in specific pathogen-free mice (Sun et al., 2021). Recent studies have focused on other microbial metabolites. For instance, trimethylamine N-oxide enhances BBB resilience to inflammation through annexin A1-mediated TJ stabilization (Hoyles et al., 2021), while in AD models, SCFAs improve TJ localization, reduce Aβ deposition, and modulate microglial phenotypes (Xie et al., 2023a). Importantly, suppressing microglial overactivation ameliorates BBB impairment in PD models, where TJ proteins are compromised (Ruan et al., 2022). These findings identify microbial metabolites as potential therapeutic targets for NDDs.

Apart from the previously discussed mechanisms, extracellular vesicles (EVs) have attracted considerable attention for their crucial role in mediating intercellular communication beyond the CNS (Xiao et al., 2021). These nano- to micro-scale lipid bilayer vesicles protect bioactive molecules from degradation and deliver them to local or distant targets, thereby facilitating communication between cells (van Niel et al., 2022). Due to their low immunogenicity, high modifiability, and ability to effectively cross the BBB (Izquierdo-Altarejos et al., 2024), extracellular vesicles are considered promising carriers for drug delivery, particularly for intracerebral applications.

Currently, extracellular nanovesicles are generally categorized into three types: exosomes derived from eukaryotic cells, outer membrane vesicles (OMVs) from Gram-negative bacteria, and membrane vesicles from Gram-positive bacteria (Çelik et al., 2022). Of particular relevance are the EVs secreted by gut bacteria, which have emerged as novel mediators within the gut–brain axis. These bacterial EVs can reach the host brain through circulatory or neural pathways (Wei et al., 2020; Zhao et al., 2021a). *Helicobacter pylori* (*Hp*) EVs can breach the BBB and trigger neuroinflammation via glial cell activation and complement pathway signaling, potentially exacerbating amyloidogenic processes (Park and Tsunoda, 2022; Xie et al., 2023b). Studies have shown that *Hp* OMVs, whether administered orally or injected, can enter the murine brain, inducing astrocyte reactivity and neuronal damage. *In vitro* experiments demonstrate that OMVs activate the NF-κB pathway, prompt astrocytes to release neurotoxic factors, inhibit neurite outgrowth, and harm neurons, suggesting that *Hp* may influence the CNS through OMVs that traverse epithelial barriers. Furthermore, other bacterial species can produce EVs with beneficial effects. For example, *Akkermansiamuciniphila* and its EVs regulate gut microbiota composition, improve intestinal barrier function, reduce inflammation, and promote metabolic homeostasis, significantly lowering body weight and adiposity in high-fat diet-fed mice (Ashrafian et al., 2019).

## Neurodegenerative Diseases and Gut Microbiota

NDDs are characterized by pathologies in which the CNS, including the brain and spinal cord, undergoes progressive degeneration of specific neuronal populations, dysfunction of glial cells, and selective disruption of synaptic networks. These interrelated pathological changes ultimately lead to irreversible neurological impairment (Sochocka et al., 2019). The selective loss of neuronal populations in the CNS results in functional deficits, including cognitive decline, emotional and personality disturbances, and loss of motor coordination. Moreover, the pathological changes occurring throughout the body, beyond the CNS, are significant in the progression of NDDs. Notably, gut microbiota dysbiosis may exacerbate neurodegenerative processes through the bidirectional communication of the gut-brain axis. The sections below will systematically explain the specific connections between NDDs and the imbalance of gut microbiota, as well as the underlying molecular mechanisms.

### Parkinson’s disease

**Figure 5:**
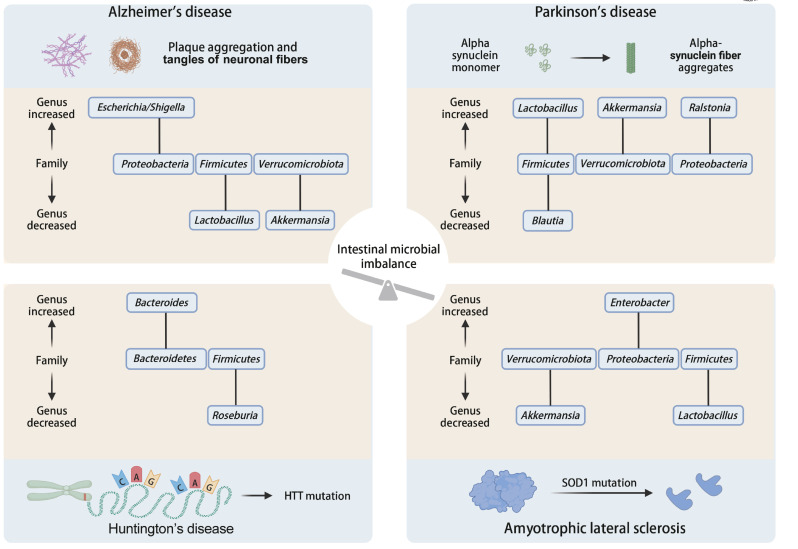
Role of gut microbiota dysbiosis in neurodegenerative diseases. Neurodegenerative diseases, including Alzheimer’s disease, Parkinson’s disease, Huntington’s disease, and amyotrophic lateral sclerosis, are closely associated with dynamic changes in the gut microbiota. The microbial composition and dominant taxa undergo considerable alterations under pathological conditions, leading to dysbiosis. This dysbiosis may inlufluence the central nervous system directly or indirectly through various mechanisms, including microbial metabolite secretion, immune regulation, and gut–brain axis signaling. These pathological processes can promote the mutation, aggregation, and deposition of aberrant proteins in neurodegenerative disorders, ultimately driving their onset and progression. Created with BioRender.com. HTT: Huntingtin gene; SOD1: superoxide dismutase 1.

PD ranks as the second most common neurodegenerative disorder worldwide (Lebouvier et al., 2009). Its clinical features primarily include bradykinesia, rigidity, resting tremor, hypokinesia, and cognitive dysfunction (Alexander, 2004). The development of PD is mainly caused by the misfolding and abnormal aggregation of α-syn, leading to the formation of Lewy bodies, which trigger neuroinflammatory responses and dopaminergic neuron loss in the midbrain (**Figure 5**; Zhou et al., 2000; Theodore et al., 2008). Notably, α-syn undergoes post-translational modifications, with phosphorylation at the S129 site significantly promoting its pathological aggregation in the brain tissues of patients with PD (Fujiwara et al., 2002).

These pathologies in the CNS show a close connection with peripheral gastrointestinal disruptions. Clinical studies have shown that patients with PD often encounter gastrointestinal dysfunction. A study that included 94 patients with PD reported that around 80% of them experienced constipation, which occurred before the appearance of motor symptoms (Ueki and Otsuka, 2004). Further research has indicated that the gut microbiota composition in patients with PD undergoes considerable alterations, along with increased intestinal barrier permeability (Mulak and Bonaz, 2015). This disruption is closely related to abnormal α-syn accumulation in the gastrointestinal mucosa (Forsyth et al., 2011), providing key evidence for the role of the gut-brain axis in PD pathogenesis. Chen et al. (2018a) confirmed that phosphorylated α-syn at the S129 site progressively accumulated in the colonic submucosa with age, leading to Parkinsonian symptoms in 9-month-old mice. Xiang et al. (2024) uncovered the molecular mechanism of pathological protein transmission between the gut and brain in PD. The α-syn and Tau proteins that accumulate in the gut can be transported to the CNS via the vagus nerve, where they are cleaved by asparagine endopeptidase into Syn N103 and Tau N368 fragments. These pathological fragments not only appear in the gut but also accumulate in the brain, accompanied by a considerable reduction in dopaminergic neurons. This discovery provides new insights into the pathological progression of PD.

While the gut–to–brain transmission of pathological proteins offers a compelling explanation for PD pathogenesis, emerging evidence suggests that the gut microbiome itself may independently or synergistically contribute to disease development. One study compared how acute (multiple MPTP injections in a single day) *versus* subchronic (single daily MPTP injections over consecutive days) administration regimens affect gut microbiota in a PD mouse model. The findings revealed distinct dysbiosis patterns associated with each regimen: in the subchronic group, both *Firmicutes* and *Bacteroidetes* were significantly increased, whereas the acute group showed no significant alteration in *Firmicutes* but a decrease in *Bacteroidetes* (Aktas, 2023). At the *Verrucomicrobia* phylum level, the acute group exhibited increased abundance, while the subchronic group demonstrated reduced levels. More importantly, fecal samples from patients with PD show a reduction in anti-inflammatory bacteria, such as *Blautia*, *Coprococcus*, and *Roseburia*, while pro-inflammatory *Proteobacteria* (*Ralstonia*) are increased (Keshavarzian et al., 2015). A recent metagenomic study of patients with PD identified elevated levels of *Lactobacillus fermentum*, *Lactobacillus reuteri*, *Lactobacillus gasseri*, *Lactobacillus rhamnosus*, and *Lactobacillus salivarius* (**Additional Table 1**; Wallen et al., 2020). Preclinical and clinical interventions targeting these dysbiosis changes have begun to reveal promising therapeutic avenues. In the MPTP-induced PD mouse model, Ishii et al. (2021) demonstrated that oral administration of *Bifidobacterium* improved cognition. Similarly, probiotic supplementation containing *Bifidobacterium*, *Lactobacillus acidophilus*, *Lactobacillus fermentum*, and *Lactobacillus reuteri* alleviated some symptoms in patients with PD (Tamtaji et al., 2019).

**Additional Table 1:** Gut microbiome alterations in Parkinson's disease

Clinical statistics	Sample	Analysis method	Microbiota alteration	Reference
66 patients with PD and 34 controls	Fecal	16S rRNA gene sequencing	↓ *Blautia, Coprococcus*, and *Roseburia ↑ Ralstonia*	Keshavarzian et al., 2015
56 patients with PD and 87 controls	Fecal	16S rRNA gene sequencing	↓ *Lachnospiraceae* and *Butyricoccus Akkermansia*	Boertien et al., 2022
54 patients with PD, 26 patients with IBD, and 16 controls	Fecal	Metagenomic sequencing	↓ *Roseburia intestinalis, Faecalibacterium prausnitzii, Anaerostipes hadrus*, and *Eubacterium rectale*	Krueger et al., 2025
20 MPTP-induced PD mice and 20 control mice	Fecal	16S rRNA gene sequencing	↓ *Lachnospiraceae, Proteobacteria ↑ Prevotellaceae, Akkermansia*	Lai et al., 2018
ASO mice and WT control mice	Fecal	16S rRNA gene sequencing	↓ *Lachnospiraceae* and *Butyricicoccus*	Sampson et al., 2016
MPTP-induced PD mouse models	Fecal	16S rRNA gene sequencing	↓ *Clostridiales ↑ Turicibacterales* and *Enterobacteriales*	Sun et al., 2018

ASO: Alpha-synuclein overexpressing; IBD: inflammatory bowel disease; MPTP: 1-methyl-4-phenyl-1,2,3,6-tetrahydropyridine; PD: Parkinson's disease; WT: wild type.

In parallel with proteinopathy, neuroinflammation constitutes a central pillar of PD pathogenesis, closely linked to non-motor symptoms that not only profoundly impair patients’ quality of life but also frequently precede the onset of motor dysfunction (Forloni et al., 2021). In α-syn-overexpressing mouse models, microglia depletion has been shown to ameliorate PD-related phenotypes (Zhang et al., 2025b). Critically, neuroinflammation in PD may originate from peripheral inflammatory triggers, with the gut emerging as a key site of initial pathology. Studies involving rodent models have shown that gut inflammation, characterized by impaired intestinal TJs, is among the earliest pathological changes in PD. When the intestinal barrier is compromised, the body is exposed to inflammatory microbial products such as LPS, leading to gut inflammation, microglial activation, neuroinflammation, oxidative stress, and ultimately pathological α-syn aggregation in neurons (Aho et al., 2021; Yang et al., 2022). This gut–brain inflammatory axis culminates in systemic immune activation, as reflected in the biomarker profiles of patients with PD. A substantial body of evidence shows elevated levels of inflammatory mediators in the brains, cerebrospinal fluid, and peripheral blood of patients with PD, including IL-1β, IL-2, IL-6, IL-10, TNF-α, chemokine ligand 5, and C-reactive protein (Chen et al., 2018b; Lin et al., 2019). Motor and cognitive impairments in patients with PD are associated with an increase in pro-inflammatory bacteria and a decrease in SCFA-producing microbiota (Ren et al., 2020; Toh et al., 2022).

Gut microbial metabolites play a critical role in PD pathogenesis. For instance, SCFAs stimulate the transcription of anti-inflammatory cytokine-encoding genes, exerting anti-inflammatory and neuroprotective effects through the gut–brain axis (Canani et al., 2011). The levels of butyrate-producing bacteria (e.g., *Prevotellaceae*, *Blautia*, *Coprococcus*, *Roseburia*) and *Faecalibacterium* in the feces and mucosa of patients with PD have been significantly reduced (Scheperjans et al., 2015). The reduction in *Prevotellaceae* abundance and the increase in *Lactobacillaceae* abundance are linked to decreased ghrelin concentrations (Dicks, 2022). Ghrelin is an intestinal hormone critical for maintaining normal DA function, and patients with PD exhibit altered ghrelin secretion (Unger et al., 2011). Alterations in microbial composition can disrupt host lipid biosynthesis and secretion pathways, including those that regulate DA production, a critical factor in PD pathogenesis, as many PD symptoms are associated with reduced DA levels (Xicoy et al., 2019). Interestingly, certain gut bacteria encode enzymes capable of synthesizing DA (Hamamah et al., 2022), further strengthening the link between microbial-derived metabolites and PD progression. Overall, these findings position the microbiota-gut-brain axis as a pivotal mediator of PD pathogenesis.

### Alzheimer’s disease

AD is a progressive neurodegenerative disorder characterized by cognitive decline and synaptic dysfunction, with pathological changes primarily affecting the hippocampus and cerebral cortex (Hayden and Teplow, 2013). Traditionally, the presence of the BBB was thought to limit the effect of external factors on AD pathogenesis. Several hypotheses have been proposed to explain the onset and progression of AD, including neuroinflammation, tau hyperphosphorylation, amyloid plaques, neurofibrillary tangles, and oxidative stress (Long and Holtzman, 2019; Zhan et al., 2023). However, its underlying mechanisms remain incompletely understood (**Figure 5**).

In recent years, accumulating evidence has highlighted the microbiota–gut–brain axis as a potentially significant factor in the development of AD (Brandscheid et al., 2017). Human studies reveal that patients with AD exhibit an overall reduction in gut microbiota abundance and diversity (**Additional Table 2**), characterized by decreased *Firmicutes* and *Bifidobacteria* alongside increased *Bacteroidetes* (Ferreiro et al., 2023). Recent reports indicate that *Coprococcus* levels are reduced in multiple brain-related disorders, including attention deficit hyperactivity disorder, autism spectrum disorder, schizophrenia, AD, PD, major depression, and bipolar disorder (Eicher and Mohajeri, 2022). Microbial imbalances are closely linked to neuroinflammation and amyloid pathology in AD. It has been reported that patients with cognitive impairment and cerebral amyloidosis display an imbalance between pro-inflammatory and anti-inflammatory gut bacteria, evidenced by an increased relative abundance of pro-inflammatory bacteria such as *Escherichia/Shigella* and a decreased relative abundance of anti-inflammatory bacteria such as *E. rectale* (Cattaneo et al., 2017). Studies using the well-established 3×Tg-AD mouse models have revealed considerable gut microbiome alterations compared with wild-type mice. Specifically, the genus *Coprococcus* markedly decreased (Ling et al., 2021), whereas the abundances of *Escherichia/Shigella* and *Barnesiella* increased (D’Argenio et al., 2022). These changes align with a pro-inflammatory state and the pathological features of AD (D’Argenio et al., 2022).

**Additional Table 2:** Gut microbiome alterations in Alzheimer's disease

Clinical statistics	Sample	Analysis method	Microbiota alteration	Reference
49 patients with AD and 115 controls	Fecal	Metagenomic sequencing	↓ *Firmicutes ↑ Bacteroidetes*	Ferreiro et al., 2023
25 patients with AD and 25 controls	Fecal	16S rRNA gene sequencing	↓ *Firmicutes* and *Actinobacteria (Bifidobacterium) ↑ Bacteroides*	Vogt et al., 2017
31 patients with MCI and 65 controls	Fecal	16S rRNA gene sequencing	↓ *Ruminococcus, Butyricimonas*, and *Oxalobacter*	Fan et al., 2023
5xFAD mice, and non-Tg WT controls	Colonic flushing	16s rRNA qPCR analysis	↓ *Bifidobacterium, Lactobacillus*, and *Firmicutes*	Shukla et al., 2021
3xTg-AD mouse model	Fecal	16S rRNA gene sequencing	↓ *Coprococcus ↑ Escherichia/Shigella* and *Barnesiella genera*	D'Argenio et al., 2022

AD: Alzheimer's disease; MCI: mild cognitive impairment; non-TgWT: non-transgenic wild type.

The human gut microbiome can induce neuroinflammatory responses in the brains of patients with AD by producing pro-inflammatory cytokines and bacterial metabolites that enter the bloodstream and cross the BBB (Giau et al., 2018). Once activated, microglia release pro-inflammatory cytokines such as IL-1β, TNF-α, and IL-6, further exacerbating neuronal damage and contributing to the progression of AD (Liang et al., 2024). LPS promote inflammatory processes, leading to amyloid deposition and tau-related pathological changes (Vogt et al., 2017). When rats are given an intraperitoneal injection of LPS, levels of inflammatory factors such as IL-1, IL-6, and TNF-α elevate, indicating that the microbiome initiates the innate immune response in AD (Zhu et al., 2014). Beyond systemic inflammation, emerging evidence suggests that gut-derived pathological proteins and microbial metabolites may directly contribute to AD pathology. Studies indicate that Aβ and tau fibril pathology may originate in the gut, where reduced levels of SCFAs influence neuroinflammatory responses and cognitive function by activating pathways such as C/EBP-β/AEP (Chen et al., 2018b, 2021a; Xia et al., 2023). Among SCFAs, butyric acid plays a key role as an energy source for colon epithelial cells and exerts immunomodulatory and anti-inflammatory effects. Another study involving patients with AD found an increase in pro-inflammatory bacteria and a reduction in bacteria capable of synthesizing butyric acid (Haran et al., 2019). In addition to butyric acid, propionic acid levels are also considerably lower in patients with AD. Propionic acid can interfere with DRP1-mediated mitochondrial fission via GPR41 and enhance PINK1/PARKIN-mediated mitochondrial autophagy through GPR43, thereby improving cognitive function in AD mouse models. Supplementing with propionic acid or propionic acid-producing *Akkermansia* helps maintain mitochondrial homeostasis (Wang et al., 2025).

### Amyotrophic lateral sclerosis

ALS is a devastating, currently incurable neurodegenerative disorder characterized by a gradual loss of motor function, leading to problems with movement, speech, and swallowing (Obrenovich et al., 2020). This condition typically results in respiratory failure and death within 2 to 5 years after onset (Gonzalez-Bermejo et al., 2016; Goutman et al., 2022). The pathogenesis of ALS involves multiple mechanisms, including protein misfolding, genetic mutations, mitochondrial dysfunction, impaired autophagy, neuroinflammation, RNA dysregulation, altered axonal transport, and gut microbiota dysbiosis (Taylor et al., 2016; Hounoum et al., 2017).

Among these pathogenic mechanisms, the genetic mutations that occur in key enzymes have been extensively studied. A key enzyme implicated in ALS is superoxide dismutase 1 (SOD1), which maintains redox homeostasis by converting superoxide radicals in the cytoplasm and mitochondria (**Figure 5**). *SOD1* mutations can induce protein misfolding and cellular stress responses, exerting neurotoxic effects on neurons and glial cells, contributing to ALS pathogenesis (Bunton-Stasyshyn et al., 2015; Kaur et al., 2016). While genetic factors are crucial, recent research has uncovered systemic interactions beyond neuronal cells, particularly through the gut–brain axis. Cox et al. (2022) demonstrated that low-dose antibiotic administration in SOD1 mice worsens motor dysfunction and reduces survival. Antibiotics deplete beneficial microbiota (e.g., *Akkermansia* and butyrate producers), downregulate microglial homeostasis genes, and upregulate neurodegenerative genes. This study underscored the protective role of the microbiota via MGnD microglia modulation, offering novel insights into gut-microglia interactions in neurological disorders. Remarkably, supplementing *Akkermansia* in SOD1 transgenic mice raises nicotinamide levels and alleviates ALS symptoms (Blacher et al., 2019). Furthermore, gut microbiota intervention improved mitochondrial function and oxidative stress pathways in SOD1 G93A mice.

Further investigations in ALS mouse models revealed considerable gut barrier dysfunction, as evidenced by reduced expression of TJ proteins (e.g., ZO-1, E-cadherin) and increased intestinal permeability (Wu et al., 2015). The accumulation of pathological SOD1 in enteric neurons has been linked to compromised gut barrier function, as indicated by decreased expression of the TJ proteins claudin-1 and occludin, as well as increased levels of the pro-inflammatory cytokines TNF-α and IL-1β (Xin et al., 2024). This mechanistic understanding is corroborated by clinical observations of microbial signatures in patients with ALS. Recent clinical studies involving 28 participants (16 patients with ALS and 12 healthy controls) identified distinct microbial alterations in patients with ALS, including increased *Enterobacter*, *Clostridium*, and *Veillonella*, alongside decreased *Prevotella* and *Lactobacillus* (Fontdevila et al., 2024). Notably, spinal ALS showed reduced propionate levels despite stable overall SCFA concentrations (Fontdevila et al., 2024). These findings emphasize the therapeutic potential of microbiota modulation in ALS. For example, *Lactiplantibacillus plantarum* promotes gut homeostasis, increases SCFA production, and demonstrates neuroprotective effects in various neurological disorders (Di Chiano et al., 2024).

### Huntington’s disease

HD is a progressive neurodegenerative disorder influenced by genetic susceptibility, environmental factors, and metabolic imbalances. It is caused by a mutation in the Huntingtin (*HTT*) gene, which involves an abnormal repetition of CAG DNA sequences, known as CAG repeats, leading to pathological changes in the huntingtin protein (**Figure 5**; Tabrizi et al., 2020). Clinically, HD is characterized by dance-like involuntary movements, progressive cognitive dysfunction, and psychiatric symptoms (Shannon et al., 2012). As the disease advances, patients may lose motor function, develop speech deficits, and eventually become bedridden (Kim et al., 2021).

Building on the clinical and genetic understanding of HD, recent studies have increasingly focused on the gut-brain axis, particularly on how intestinal dysbiosis (i.e., an imbalance in the gut microbiota) may contribute to HD pathogenesis. In the HD transgenic mouse model R6/1, Kong et al. (2020) observed clinical symptoms of HD and aggregates of mutant huntingtin, coupled with increased *Bacteroides* and decreased *Firmicutes* (Kong et al., 2020; Stan et al., 2020). These changes were associated with elevated intestinal permeability, inflammation, and motor deficits. These findings in animal models have been further supported by human studies, where researchers collected stool and serum samples from patients with HD and healthy controls for metagenomic and metabolomic analyses. Their results showed that, at the phylum level, the gut microbiota of patients with HD was predominantly composed of *Firmicutes* and *Bacteroidetes*, while at the genus level, *Bacteroides* and *Faecalibacterium* were increased, and SCFA-producing bacteria such as *Lachnospira* and *Roseburia* were considerably reduced (Qian et al., 2024).

Beyond microbial composition, the metabolic consequences of gut dysbiosis in HD have also been explored. Mutations in the *HTT* gene also induce mitochondrial dysfunction, which plays a part in the pathogenesis of HD (Tabrizi et al., 2020). HD mice exhibit remrakably elevated ATP levels in plasma compared with controls, suggesting that gut microbes may modulate plasma metabolite levels along the gut–brain axis (Kong et al., 2021). For instance, SCFAs produced by gut bacteria can stimulate colonic cells to create more ATP, which is then released into the bloodstream. This kind of metabolic dysregulation is further compounded by alterations in tryptophan metabolism. In patients with HD, increased activity of indoleamine 2,3-dioxygenase results in a higher kynurenine/tryptophan ratio, while decreased kynurenine aminotransferase activity leads to the buildup of neurotoxic metabolites. This drives mitochondrial dysfunction and oxidative stress, which is mediated by N-methyl-D-aspartate receptors (Stoy et al., 2005).

Additionally, the role of microbial-derived neurotransmitters in the pathogenesis of HD has been receiving increasing attention. For example, previous studies have reported decreased levels of GABA, a metabolite produced by *Lactobacillus reuteri*, in the brains of patients with HD (Perez-Rosello et al., 2019; Barry et al., 2020). Recent studies have clarified that GABA modulates macrophage-mediated inflammatory responses through dual mechanisms. The synergistic action of these two pathways significantly suppresses IL-1β secretion in macrophages and effectively alleviates both sepsis and metabolic inflammation in animal models (Fu et al., 2022). Moreover, the interplay between gut dysbiosis and neuroinflammation in HD has been further elucidated. In the BV2 microglial cell line, overexpression of mutant huntingtin (mHTT) spontaneously increases the expression of pro-inflammatory genes such as IL-6 and TNF-α, even in the absence of external inflammatory stimuli (Crotti et al., 2014). Finally, the integrity of the gut–brain barrier has emerged as a critical factor in HD progression. Further evidence from R6/2 mice shows a deficiency of the TJ protein occludin in both the BBB and colonic mucosa, as well as in damaged epithelial tissues. This suggests that alterations in gut microbiota may be related to neuronal toxicity in HD (Stan et al., 2020).

### Multiple sclerosis

MS is a chronic immune-mediated disorder characterized by inflammatory demyelination in the CNS. Two key clinical features define this disease: spatial dissemination (multifocal neurological impairments) and temporal heterogeneity (a relapsing-remitting course). MS pathophysiology involves a complex interaction between inflammatory activity and neurodegenerative processes, eventually leading to progressive neurological deterioration. Even though the CNS is the primary disease target, emerging evidence suggests that peripheral immune dysregulation, particularly via the gut-brain axis, plays a pivotal role in MS pathogenesis.

Patients with MS display alterations in the gut microbiota that trigger autoimmune responses. Notable changes include an increase in *Methanobrevibacter* and *Akkermansia* species, along with a decrease in *Butyricimonas*, which correlate with abnormal dendritic cell maturation and dysregulated interferon signaling and NF-κB pathway gene expression in peripheral immune cells (Jangi et al., 2016). A recent comparative study also reported that untreated patients with MS exhibit reduced levels of *Actinobacteria*, *Bifidobacterium*, and *Faecalibacterium*, along with elevated *Prevotella*, compared with healthy controls (Vacaras et al., 2023). Of particular clinical importance is that SCFA-producing bacteria, such as *Eubacterium hallii*, *Butyricicoccus*, and *Blautia*, have been shown to decrease significantly in deteriorating patients with MS (Schwerdtfeger et al., 2025). Further evidence from monozygotic twin studies reveals that increased *Akkermansia* abundance in patients with MS promotes autoimmune responses. Fecal microbiota transplantation from these patients into mouse models increases disease incidence and reduces IL-10 secretion (Berer et al., 2017).

Experimental autoimmune encephalomyelitis (EAE) model studies have clarified specific gut microbiota mechanisms in regulating neuroinflammation. Germ-free mice exhibit much milder EAE symptoms, accompanied by reduced IL-17A levels and increased regulatory T-cell populations in both the gut and the CNS. Surprisingly, colonization with segmented filamentous bacteria alone can induce EAE by promoting Th17 cell accumulation in the CNS. These findings provide critical insights into the gut-brain axis in MS pathogenesis and highlight potential therapeutic targets (Lee et al., 2011).

### Frontotemporal dementia

Frontotemporal dementia (FTD) is a neurodegenerative disorder characterized by progressive degeneration of the frontal and temporal lobes, clinically manifesting as behavioral abnormalities, executive dysfunction, and language impairment (Swift et al., 2021). Accounting for 5%–15% of all dementia cases (Joshi et al., 2023), FTD primarily includes behavioral variant FTD and primary progressive aphasia, with its pathological basis typically associated with abnormal protein deposition linked to frontotemporal lobar degeneration. As the second most common early-onset dementia, and with limited treatment options, FTD represents a critical area requiring further research into pathological mechanisms and therapeutic development.

Mendelian randomization analysis was used to reveal, for the first time, potential causal relationships between gut microbiota, cytokines, and five dementia subtypes (Ji et al., 2024). Notably, Ji et al. (2024) identified that the genus *Desulfovibrio* may exert protective effects against FTD. However, current research on the gut–brain axis in FTD remains at an early stage, necessitating additional clarification of causal relationships between gut microbiota and cerebral pathological proteins, such as tau, TDP-43 (San Gil and Walker, 2025), as well as subtype-specific mechanisms (such as behavioral variant and primary progressive aphasia). Future studies should integrate multi-omics technologies to elucidate the role of the gut-brain axis in FTD pathogenesis, providing scientific foundations for developing precision interventions.

## Safety of Gut Microbiome–Related Therapies for Neurodegenerative Diseases

Interventions targeting the gut microbiota in NDDs, including probiotics, prebiotics, fecal microbiota transplantation, SCFA supplements, GLP-1 receptor agonists, and immunomodulators, have demonstrated therapeutic potential, but their safety profiles and associated adverse effects warrant close attention.

In terms of adverse reactions, probiotics (e.g., *Lactobacillus* and *Bifidobacterium* preparations) are generally well-tolerated, though some patients may experience transient gastrointestinal disturbances such as bloating, diarrhea, constipation, or abdominal discomfort. Notably, studies have confirmed that the use of Bifidobacterium breve probiotics leads to bacteremia in neonates, primarily triggered by intestinal pathologies like perforation or enteritis. Although blood culture positivity may take longer to detect, clinical manifestations are typically mild, with favorable outcomes following antibiotic treatment. These findings highlight the need for vigilance regarding probiotic-associated bacteremia risks, particularly in neonates with intestinal damage (Sakurai et al., 2022).

FMT is widely regarded as safe in clinical practice, with common adverse effects being mild and transient gastrointestinal symptoms, including short-lived diarrhea, mild abdominal cramping, low-grade fever, bloating, and increased flatulence. However, as an invasive therapy for recurrent or refractory *Clostridioides difficile* infection, FMT has been associated with infectious complications (DeFilipp et al., 2019). Safety assessment requires a multidimensional integration of indicators: preclinical studies should utilize animal models to evaluate acute/chronic toxicity, reproductive toxicity, and carcinogenicity. During clinical trials, key monitoring parameters include vital signs, laboratory tests (complete blood count, liver/kidney function, and inflammatory cytokine profiles), dynamic changes in gut microbiota, and fluctuations in neurological symptoms.

From a regulatory perspective, the U.S. FDA classifies fecal microbiota transplantation as a biological product requiring strict donor screening and standardized preparation protocols, while probiotics are generally regulated as dietary supplements, leading to variations in quality control standards. Importantly, individual differences (such as age, underlying conditions, and intestinal barrier function) remarkably influence safety outcomes; for instance, older adult patients with increased intestinal permeability face higher risks of microbial metabolite or pathogen translocation into the bloodstream.

Current safety research on gut microbiota-targeted therapies for NDDs remains in the data accumulation phase, with most evidence derived from short-term trials or case reports. The long-term safety profile, particularly chronic effects on the BBB and neurotransmitter balance, requires urgent in-depth investigation. Future efforts should focus on establishing a unified safety assessment system integrating microbiome sequencing, blood biomarker monitoring, and neurological function scales to dynamically evaluate intervention risk-benefit ratios. Special attention must be given to immunocompromised populations and patients taking concomitant medications (such as immunomodulators and anti-PD drugs) to ensure safety for precision medicine applications.

## Clinical Translation

In recent years, research on the microbiota-gut–brain axis has provided novel therapeutic approaches for NDDs (**Additional Table 3**). Numerous clinical studies have demonstrated that specific probiotic strains (e.g., *Bifidobacterium longum subsp. infantis* BLI-02, *Lactobacillus plantarum* PL-02) can exert beneficial effects on AD and PD through multiple mechanisms, including modulation of neurotrophic factors (such as 36% increase in BDNF levels) (Hsu et al., 2023), attenuation of neuroinflammation (reductions in IL-1β and TNF-α), and improvement of oxidative stress (enhanced SOD activity). Notably, combined probiotic and vitamin D interventions have shown synergistic effects in patients with PD, remarkably improving inflammatory markers and oxidative stress indicators while also alleviating gastrointestinal and neuropsychiatric symptoms (Zali et al., 2024). Furthermore, synbiotic formulations containing specific strains (e.g., *Lacticaseibacillus paracasei* DG) have been shown to significantly enhance autonomic nervous function and emotional status in patients with PD by increasing the abundance of beneficial bacteria such as *Faecalibacterium prausnitzii* (Andreozzi et al., 2024).

**Additional Table 3:** Clinical research on microbiome-based therapeutics for neurological disorders

Registration ID	Sample size	Key point	Reference
NCT04685252	Prenatal intervention group (*n* = 62) Postpartum intervention group (*n* = 61) Placebo control group (*n* = 61)	(1) Probiotics ineffective for perinatal mood: A RCT (*n* = 184) found *B. longum* NCC3001 did not improve mood (EPDS/STAI) compared with placebo, with low detection in some participants. (2) More research needed: Study highlights uncertainty in microbiome-mood links for perinatal health.	Fries et al., 2025
NCT 06496438	Psychobiotics group (*n* = 146) Placebo group (*n* = 149)	(1) Psychobiotics alleviate distress in patients with cancer: post-operative patients with gastrointestinal cancer experienced reductions in depression (60.4%), anxiety (57.0%), and stress (60.4%) with psychobiotics, while those receiving placebo exhibited worsening symptoms. (2) Promising adjunct to cancer care: Results support the role of psychobiotics in managing treatment- related mental health challenges, highlighting their potential as a new supportive therapy.	Tzikos et al., 2025
NCT04199845	PS128 group (*n* = 16) Placebo group (*n* = 16)	(1) No antidepressant effect found: *L. plantarum* PS128 did not alleviate depression symptoms, inflammation, or gut flora in 32 patients with MDD after 8 weeks compared with placebo. (2) Require further investigation: Although minor microbial changes were observed, larger studies are needed to assess the potential of psychobiotics for MDD.	Lin et al., 2024
NCT04293159	Patients with constipated PD (*n* = 30)	(1) Synbiotic aids for patients with PD: *L. paracasei* DG combined with inulin alleviated constipation, non-motor symptoms, and gut flora (*Oscillospirales*, *F. prausnitzii*) in 30 patients with PD after 12 weeks. (2) Gut-brain connection: Show promise for managing PD symptoms through microbiome modulation.	Andreozzi et al., 2024
NCT05145881	Active control group (*n* = 30) Treatment group (*n* = 30)	(1) Probiotics enhance brain biomarkers: High-dose probiotics increased BDNF levels by 36% and reduced inflammation in patients with AD, demonstrating gut-brain effects. (2) Cognitive impact unconfirmed: Despite the presence of neuroprotective changes, no clear cognitive benefits were observed.	Hsu et al., 2023

AD: Alzheimer's disease; BDNF: brain-derived neurotrophic factor; EPDS: Edinburgh Postnatal Depression Scale; MDD: major depressive disorder; NCT: National Clinical Trial; PD: Parkinson's disease; RCT: randomized controlled trial; STAI: State-Trait Anxiety Inventory.

In experiments related to FMT therapy, studies have found that rotenone can induce gut microbiota dysbiosis, characterized by an increase in the bacterial genera *Akkermansia* and *Desulfovibrio*. This dysbiosis further triggers gastrointestinal dysfunction, motor deficits, and other symptoms of PD, with the process involving the pathogenesis of the disease through the microbiota–gut–brain axis. In contrast, FMT is capable of restoring the balance of the gut microbiota, alleviating intestinal inflammation and barrier damage, and reducing systemic inflammation levels. Consequently, it reduces BBB impairment and neuroinflammation in the substantia nigra, thereby minimizing damage to dopaminergic neurons. The underlying mechanism may be associated with a reduction in lipopolysaccharide levels in the colon, serum, and substantia nigra, as well as the inhibition of TLR4-related signaling pathways and their downstream pro-inflammatory products (Zhao et al., 2021b). Furthermore, experiments involving antibiotic-induced gut microbiota depletion and FMT have revealed that antibiotic-mediated microbiota depletion can enhance the bioavailability of levodopa and increase striatal dopamine levels, which are correlated with improvements in motor function. Mice that received fecal microbiota transplantation from a good responder group exhibited considerably higher levodopa bioavailability compared with those that received transplantation from a moderate responder group (Ai et al., 2025). Meanwhile, studies focusing on MS have explored the therapeutic potential of FMT for this disease. Tests conducted using EAE, a mouse model of MS, have shown that FMT can rectify gut microbiota dysbiosis to a certain extent and exert a therapeutic effect on EAE. Specifically, it can reduce the activation of microglia and astrocytes while also protecting the BBB, myelin, and axons (Li et al., 2020).

In addition to the development and application of probiotics and prebiotics, the effect of dietary structure and nutritional components on neuroinflammation is increasingly drawing attention. Intermittent fasting can exert neuroprotective effects by maintaining microbial community balance and restoring levels of microbially derived metabolites (Tanca et al., 2018). A 4-week intermittent fasting study demonstrated that it alleviates dopamine neuron degeneration, α-syn pathological aggregation, and motor dysfunction in rAAV-α-syn mouse models, with similar efficacy observed in aged mice (though weaker than in young mice). Mechanistically, intermittent fasting enhances the formation and maturation of autophagic vesicles in the substantia nigra, promotes the clearance of phosphorylated α-syn, and reduces insoluble α-syn aggregation, while regulating the transition of microglia to an anti-inflammatory phenotype and reducing neuroinflammation (Szego et al., 2025).

The Mediterranean diet is characterized by a high intake of fruits, vegetables, legumes, nuts, and olive oil, as well as moderate to high consumption of fish, moderate red wine consumption, and low intake of meat and dairy products. This diet possesses anti-inflammatory, antioxidant, and gut microbiota-regulating properties, potentially exerting neuroprotective effects. A 2006 study with an average follow-up of every 4 years found that adherence to the Mediterranean diet is closely associated with the risk of AD, with higher adherence linked to a lower risk of AD (Scarmeas et al., 2006). Another study of 42,582 Swedish women indicated that following the Mediterranean diet may reduce the risk of PD in individuals over 60 years of age and the risk of amyotrophic lateral sclerosis in those under 60 years of age, although it was not significantly associated with AD (Joyce et al., 2025).

The ketogenic diet, a high-fat, low-carbohydrate dietary pattern, stimulates the synthesis of ketone bodies to provide energy. Research shows that during postnatal development, cellular metabolism dynamically adjusts in response to nutritional changes. After weaning, glycolytic pathways are enhanced while ketolytic pathways are weakened, with particularly significant changes observed in astrocytes and oligodendrocytes (Düking et al., 2022). In an EAE model, neuroinflammation-induced neuronal metabolic remodeling aligns with the effects of the ketogenic diet, enhancing both ketolysis and the expression of energy metabolism enzymes. However, EAE cannot meet metabolic demands due to peripheral nutritional deficiency; supplementing with a ketogenic diet can alleviate inflammatory damage by increasing the supply of ketone bodies to match neuronal metabolic needs (Düking et al., 2022). Additionally, the ketogenic diet can suppress EAE by reducing Th1 and Th17 responses, decreasing reactive oxygen species production, and increasing regulatory T cell responsiveness (Choi et al., 2016). Another study found that ketogenic diet intervention can downregulate mTOR and upregulate eNOS expression in young healthy mice, increasing cerebral blood flow and enhancing the P-glycoprotein transport function of BBB to promote amyloid-β clearance, thereby improving cerebral vascular function. Concurrently, it enriches beneficial bacteria such as *Akkermansia muciniphila* and *Lactobacillus*, while reducing pro-inflammatory groups such as *Desulfovibrio* and *Turicibacter*. Early intervention helps optimize metabolic status and reduce the risk of NDDs such as AD (Ma et al., 2018). In summary, from the development of drugs targeting the gut microbiota, such as probiotics, to the clinical application of microbiome restoration techniques such as fecal microbiota transplantation, as well as the formulation and implementation of personalized dietary intervention plans, these strategies centered on the gut microbiota have demonstrated considerable potential in the prevention and treatment of NDDs. Specifically, these interventions not only regulate the homeostasis of the microbiota-gut-brain axis to effectively suppress excessive inflammatory responses in both the CNS and peripheral nervous system, thereby reducing neuronal damage and apoptosis; they also play a role in improving cognitive function in patients, such as alleviating symptoms of memory decline and cognitive slowing, and enhancing the ability to perform daily cognitive activities. Additionally, for diseases such as PD that involve motor dysfunction, such interventions can help restore motor coordination, alleviate symptoms such as tremors and rigidity, and improve patients’ motor endurance and ability to perform daily activities independently. These multifaceted positive effects provide new insights and directions for the treatment of NDDs and lay the foundation for further exploration of more precise and effective combined intervention strategies.

However, current clinical translation still faces important limitations: (1) Strain specificity issues—considerable variations exist in strain combinations and dosages across studies, leading to a lack of standardized protocols; (2) Unclear mechanisms—while changes in microbial composition correlate with clinical improvement, the causal chain of evidence remains incomplete; (3) Limited cognitive improvement—in patients with AD, although biomarker improvements are significant, cognitive enhancement often fails to reach statistical significance; (4) Significant individual variability—baseline gut microbiota composition and disease stage may influence intervention outcomes.

Current studies focus on developing precise strain combinations to address these challenges. Utilizing multi-omics techniques such as macro-genomics and metabolomics, the researchers aim to elucidate molecular mechanisms, explore synergistic effects between microbial treatments and conventional therapies, and conduct larger-scale, long-term clinical trials to assess cognitive endpoints. In addition, personalized microbial intervention strategies based on individual gut flora characteristics are emerging as a new research direction. These advances lay an important foundation for the clinical translation of microbial therapy for NDDs.

## Limitations of the Current Research

The microbiota–gut–brain axis enables two-way communication through neural, immune, and endocrine routes, offering potential avenues for the treatment and prevention of NDDs. However, translating preclinical evidence into clinical applications faces three primary obstacles: (1) limitations of animal models; (2) the unresolved complexity of the mechanisms; and (3) uncertainty regarding the causal relationship between gut and brain pathology.

First, although multiple studies have demonstrated a partial overlap in gut microbiota alterations between various mouse models and human patients (Cattaneo et al., 2017; D’Argenio et al., 2022), animal models cannot fully recapitulate the complexity or progression of human diseases, limiting the predictive value of interventions such as FMT. For instance, while FMT can repair antibiotic-induced BBB damage in mice (Sun et al., 2021), its efficacy in humans remains uncertain due to ethical constraints and inter-individual variability. Additionally, current diagnostic methods primarily rely on symptom clusters (such as abdominal pain with anxiety) rather than definitive biomarkers (such as specific microbial signatures or metabolites), contributing to high rates of misdiagnosis. Even when potential targets such as SCFAs are identified, their dose-dependent pro- and anti-inflammatory properties (Vinolo et al., 2009; Di Chiano et al., 2024) complicate therapeutic applications.

It is unclear how the vagus nerve functions within the gut–brain axis, adding to the complexity of the situation. While some researchers suggest that the vagus nerve transmits inflammatory signals that affect brain function (Borovikova et al., 2000), other studies highlight its role in regulating metabolism and appetite. Additionally, gut barrier function depends on multiple factors, including microbial metabolites, cytokines (such as TNF-α and IFN-γ), and dietary components. The significance of these factors varies considerably across different disease patterns and patient groups. Furthermore, the multifactorial pathology of NDDs such as AD—encompassing brain inflammation, mitochondrial dysfunction, and impaired synaptic function (Long and Holtzman, 2019)—complicates the identification of the primary factor driving disease progression.

Lastly, it remains unclear whether the α-syn deposits observed in the intestines of patients with PD (Forsyth et al., 2011) precede the emergence of brain pathology or follow it. This lack of certainty highlights a broader discussion in biology regarding whether the pathological alterations in NDDs originate in the gut and subsequently spread to the brain, or first manifest in the CNS and then extend to peripheral organs.

Despite these challenges, research on the microbiota–gut–brain axis holds significant potential. Continuous breakthroughs in understanding microbe-host interactions are revealing novel therapeutic targets. Microbial metabolites, such as SCFAs and tryptophan derivatives, along with their receptors (such as G protein-coupled receptors), have emerged as promising therapeutic targets (Lorente et al., 2025). Leveraging these insights, synthetic biology may radically transform delivery methods. In the future, engineered probiotics designed to release neuroprotective factors or degrade pathogenic proteins could function as “living drugs,” enabling a targeted and more efficient route of administration via the gut. To realize this potential, collaboration across various disciplines is essential. Specifically, we need to create a comprehensive gut–brain axis database that integrates metagenomic, metabolomic, microbiome, and neuroimaging data. Such a resource would be critical for advancing precision diagnostic tools and treatments for NDDs.

Apart from the research limitations mentioned above, this review article has its own limitations in comprehensively addressing the topic and proposing solutions. While it highlights key challenges in microbiota–gut–brain axis research, it may not fully cover the wide variety of existing studies and emerging theoretical frameworks. As a result, it could overlook important insights that would enrich the discussion. Additionally, the analysis of complex mechanisms remains insufficiently detailed, particularly regarding the specific physiological and genetic differences between animal models and humans, as well as the intricate interactions among microbial metabolites, immune mediators, and neural pathways. Furthermore, although the article acknowledges research prospects such as targeted therapies and interdisciplinary collaborations, it does not provide concrete methodologies for overcoming translational hurdles, such as defined protocols for constructing integrated gut-brain axis databases or structured frameworks to facilitate cross-disciplinary cooperation. These omissions indicate that a more detailed discussion is needed to bridge the gap between current limitations and workable solutions.

## Conclusions

This review emphasizes that the gut-brain axis regulates the network driving the pathological progression of NDDs through a multi-dimensional “microbe-host-neuro-circulation.” This process includes the regulation of intestinal microbiota, vagus nerve signal transduction, and gut-derived systemic circulation. SCFAs and tryptophan derivatives can dynamically regulate inflammatory pathways from the periphery to the CNS by maintaining the integrity of the intestinal barrier, activating intestinal ion channels, and preserving local immune homeostasis. The vagus nerve, as a bidirectional signaling center, not only transmits metabolic signals from the intestinal flora to the CNS but also regulates intestinal barrier permeability and microbial balance through the cholinergic anti-inflammatory pathway. Simultaneously, the interaction between the intestinal barrier and the BBB allows microbial-derived molecules (such as lipopolysaccharides, or LPS) and immune mediators (such as TNF-α and IL-1β) to enter the systemic circulation, weakening the BBB and activating microglia. This series of events leads to neuroinflammation, accelerating degenerative pathological conditions such as amyloid-β protein deposition and α-syn aggregation. Elucidating these mechanisms reveals several promising therapeutic avenues—microbiome remodeling, vagus nerve stimulation, and barrier restoration—that may ultimately lead to effective treatments for NDDs.

## Additional files:

***Additional Table 1:*** *Gut microbiome alterations in Parkinson’s disease.*

***Additional Table 2:*** *Gut microbiome alterations in Alzheimer’s disease.*

***Additional Table 3:*** *Clinical research on microbiome-based therapeutics for neurological disorders.*

## Data Availability

*All relevant data are within the paper and its Additional files*.
